# The Effectiveness of Self-Guided Digital Interventions to Improve Physical Activity and Exercise Outcomes for People With Chronic Conditions: A Systematic Review and Meta-Analysis

**DOI:** 10.3389/fresc.2022.925620

**Published:** 2022-06-24

**Authors:** Verna Stavric, Nicola M. Kayes, Usman Rashid, Nicola L. Saywell

**Affiliations:** ^1^Rehabilitation Innovation Center, School of Clinical Sciences, Auckland University of Technology, Auckland, New Zealand; ^2^Centre for Person-Centered Research, School of Clinical Sciences, Auckland University of Technology, Auckland, New Zealand

**Keywords:** physical activity, exercise, behavioral strategies, digital, self-guided, chronic conditions, systematic review and meta-analysis

## Abstract

**Objective:**

The aim of this systematic review was to determine the effectiveness of self-guided digital physical activity (PA) and exercise interventions to improve physical activity and exercise (PA&E) outcomes for people living with chronic health conditions. Digital health interventions, especially those with minimal human contact, may offer a sustainable solution to accessing ongoing services and support for this population.

**Methods:**

A comprehensive and systematic search was conducted up to December 2021, through seven databases, for randomized trials that evaluated the effect of self-guided web- or internet-based PA interventions on physical activity or exercise outcomes. Included studies had to have interventions with minimal human contact and interaction with participants needed to be automatically generated. All studies were screened for eligibility and relevant data were extracted. Two independent reviewers assessed the risk of bias using the Cochrane risk of bias tool. Standardized mean differences and 95% confidence intervals (CI) were calculated. PA data were pooled, and forest plots were generated.

**Results:**

Sixteen studies met the eligibility criteria and included a total of 2,439 participants. There was wide variation in health conditions and intervention characteristics in mode and parameters of delivery, and in the application of theory and behavioral strategies. Self-reported PA in the intervention group was greater than controls at the end of the intervention [standardized mean difference (SMD) 0.2, 95% CI = 0.1, 0.3] and at follow up (SMD 0.3, 95% CI 0.2–0.5). The difference in objectively measured PA was small and non-significant (SMD 0.3, 95% CI −0.2 to 0.9). All interventions included behavioral strategies and ten of the sixteen were underpinned by theory.

**Conclusions:**

Self-guided digital PA&E interventions provided a positive effect on PA immediately after the intervention. An unexpected and positive finding was a sustained increase in PA at follow-up, particularly for interventions where the behavioral strategies were underpinned by a theoretical framework. Interventions with minimal contact have the potential to support sustained PA engagement at least as well as interventions with supervision.

**Systematic Review Registration:**

https://www.crd.york.ac.uk/prospero/, identifier: CRD42019132464.

## Introduction

There is extensive evidence for the benefits of physical activity (PA) in managing chronic conditions given their impact on fitness, mobility and general health (1, 2). Interventions aiming to address physical inactivity do not appear to have been implemented in any meaningful way (3). This may in part be due to the limited availability of clinic-based, face-to-face interventions (4, 5) to address the unique needs of this population. Alternative methods of delivering PA and exercise (PA&E) interventions need to be explored.

Digital technologies and the internet offer a medium to deliver and support PA&E interventions. These can be defined as interventions that are delivered via a digital platform to support or encourage a person to perform PA or exercise, usually with the aims of improved health outcomes. They provide a mode of health care delivery for people who find standard care inaccessible due to physical, economic, or social barriers (6, 7). Advancements in technology and digital content have allowed the development of digital therapeutic interventions that encourage people to use them with minimal support. These interventions have minimal to no ongoing human involvement in their set up and can be delivered automatically. Applications, incorporating behavior change elements and persuasive features (8–11), can be incorporated into devices to offer interactive and personalized approaches (12).

Previous reviews have investigated the effectiveness of digital PA interventions in the general adult population and meta-analyses have demonstrated positive effects on PA (6, 13). However, people living with chronic health conditions face unique challenges accessing and undertaking PA and exercise. They express a desire for specialist knowledge; concern that exercising may exacerbate symptoms; and transport issues (7, 14–17). A previous review by Bossen and colleagues (18) investigated the use of web-based interventions with minimal human contact, designed to increase PA in people living with chronic health conditions. They reported mixed results with no clear conclusion.

Several factors mean that an updated review is warranted. First, all studies in the review by Bossen et al. (18) were published between 2005 and 2010. Innovations in technology and increasing acceptance of its use in therapeutic interactions have led to a change in the definition of minimal human contact. Second, the review did not include exercise-based interventions. Exercise is a subcategory of PA (19) and has proven benefits in many chronic health populations (20, 21). Therefore, in this review, our reporting of PA interventions will include exercise interventions. There has been no review to date investigating digital PA&E interventions that include behavioral intervention features, for people living with chronic health conditions, delivered with minimal human contact.

The aim of this systematic review was to determine the effectiveness of self-guided digital PA&E interventions to improve PA&E related outcomes for people living with chronic health conditions. A secondary aim was to determine key behavioral intervention features that were used in the selected studies.

## Methods

The protocol was registered with PROSPERO; CRD 42019132464. The review has been conducted and is reported following the guidelines of the Preferred Reporting Items for Systematic Reviews and Meta-Analyses (PRISMA) statement Moher et al. (22).

### Eligibility Criteria

Papers were included if they met the criteria as outlined in Table 1.

**Table 1:** Eligibility criteria of papers.

**Elements**	**Inclusion criteria**	**Exclusion criteria**
Study design and reporting		
	Randomized controlled trial (RCT) or pilot that contains data addressing effectiveness	Not in English Publication not peer-reviewed Conference proceeding
	Full text available Published up to end of December 2021	
Population		
	Adults with a chronic condition defined as a human health condition or disease that is persistent or otherwise long-lasting in its effects (23)	Those at risk of developing a chronic condition Those caring for a person living with a chronic condition
Intervention		
	Designed for use by people living with a health condition Explicitly supports physical activity or exercise in a self-guided program Web-based or app-based Intervention has minimal human contact comprising no more than initial contact for set up or orientation AND Any ongoing interaction with the program is generated automatically	
Outcome		
	Any physical activity or exercise related outcome that measures a body function, an activity or a participatory limitation as per the International Classification of Functioning, Disability and Health Framework (ICF) (24)	

The key elements in Table 1 were used to devise the search strategy. Specifically, key terms were derived for “intervention” (digital exercise) and “study design” (randomized controlled trial). The search strategy did not include search terms for “population” and “outcome” to keep the reach as broad as possible. The criteria for population and outcome were applied during the screening process. Search terms were combined using Boolean, wildcard, truncation, and proximity searching. The search strategy was tailored to specific databases. Table 2 shows the search conducted using an OVID database.

**Table 2:** Search concepts and terms using OVID.

	**Search concept**	**Terms used as keyword OR title**
#1	Digital physical activity or exercise	
		(“world wide web” OR “web based” OR “web-based” OR website* OR “web site*” OR “web app*” OR internet OR online OR Ehealth OR “e-health” OR telemedicine OR telecare OR telehealth OR “tele-health” OR telerehab* OR “tele-rehab*” OR “digital health” OR mHealth OR “m-Health” OR “mobile health” OR “mobile app*” OR “smartphone app*” OR “digital intervention*”) ADJ8 (exercis* OR rehab* OR physiotherapy* OR “physical therap*” OR “physical activ*” OR “fitness train*”)
#2	Study design RCT	
		“Random* control*” OR RCT OR “control* trial*”
#3	#1 AND #2	

### Databases

Literature searches were conducted in the following databases up until the end of December 2019. This was updated to include any new publications to the end of December 2021: Cumulated Index to Nursing and Allied Health Literature (CINAHL), MEDLINE, SPORTSDiscus through EBSCO Health Database, Allied and Complementary Medicine (AMED), Evidence Based Medicine (EBM) Reviews—Cochrane Methodology, EBM Reviews—Health Technology Assessment, PsycINFO, Ovid MEDLINE(R) through OVID, Scopus, clinicaltrials.gov, Cochrane Central Register of Controlled Trial (CENTRAL), and in Web of Science. The PEDro database was searched (using simplified broad key terms). Reference lists of relevant reviews and articles were also hand searched.

### Data Extraction

All citations returned in the search were downloaded and saved into EndNote X8. Duplicates were removed and then titles screened by VS, according to the pre-defined inclusion criteria. A selection of titles (the first 100) was independently screened by a second assessor (NS). Any disagreements were reviewed and discussed to ensure consensus was reached. A third assessor (NK) acted as arbitrator. Thereafter, VS and NS would meet after every 200 titles. There was high agreement as to which titles warranted further review. This process refined selection criteria. The abstracts and then full texts of studies identified as probably meeting the inclusion criteria were reviewed by VS in consultation with NS to confirm the final set of included studies.

Key details from each of the included studies were recorded in a data extraction table in Excel. These included: author and country; participant numbers and characteristics; study design; treatment intervention parameters including duration, frequency, and follow up. The synthesis of findings from key papers in the area (25–29) were used to create a framework to guide data extraction and included: theoretical underpinning for the intervention; instruction on how to perform the PA or exercise; recording and tracking of PA or exercise; the use of goal setting; the use of action and coping planning; the type, use, and delivery of feedback and monitoring; the use and delivery of prompts; the use of any additional online PA or exercise resources; the use of PA or exercise testimonials; and the use of gamification. PA&E related outcome measures and results at the end of the intervention, and at follow up, if reported, were also recorded. If multiple impairment level outcomes were measured, only outcomes that have previously been shown to be correlated with the construct being measured (e.g., plantar flexion strength with walking) were extracted. For studies that were comparing more than two arms, data from arms comparing self-guided interventions to a control were included in the analysis.

### Risk of Bias

Risk of bias for each of the included studies was categorized as low, having some concerns or high, drawing on the revised Cochrane risk-of-bias tool for randomized trials (RoB 2) (30). Initially, two studies were scored independently by two authors (VS, NK). Scores were compared and key points of disagreement were discussed, to improve interrater agreement on interpretation of the RoB 2 criteria. Following that, all included studies were independently assessed by both authors. Rates of agreement were calculated and are reported below. Disagreements were discussed to achieve consensus, with NS acting as arbitrator.

### Data Synthesis

Data were narratively synthesized focusing on the characteristics of the studies and outcomes. Meta-analysis was conducted on all studies that used PA as an outcome. The PA outcome measures were categorized as self-report or instrumented, and the data from each were pooled separately for meta-analysis.

A summary of intervention effects for each study was obtained by calculating Hedge's g standardized mean differences (SMD)s, 95% confidence intervals (CI)s, standard deviations (SD)s and effect sizes (ES). The Hedge's g values were calculated from the post-intervention time points while accounting for the pre-intervention differences. A positive ES indicates a result in favor of the intervention and a negative in favor of the control. When insufficient data were available for analysis, study authors were contacted. If the data were not received or could not be computated from published material, it was not included in any further analysis and was noted as not reported (NR). If standard errors or confidence intervals were presented instead of standard deviations (SD), SDs were calculated using recommended formulae (31). When required, means and SD were approximated from figures using WebPlotDigitizer (32, 33).

Given the clinical heterogeneity of the included studies, both fixed effects and random-effects models were considered for pooling PA data. The extent of heterogeneity was determined using a hypothesis test based on generalized Cochran's Q-statistic (34). High heterogeneity was assumed when the *Q*-test coincided with a significant value (*p* < 0.05) (35) in which case, a random effects model was used. *I*^2^ statistic was presented if the random effects model was chosen (36). Meta-analysis results were reported as pooled Hedge's g and 95% CIs. Hedges'g ≤0.2, ≥0.5, and ≥0.8 were interpreted as small, medium, or large, respectively (37). A CI which did not overlap zero was considered statistically significant. An intervention was interpreted as effective at improving a construct when the estimated effect size was positive and had a 95% CI which did not cross zero. A category of interventions was considered effective at improving a construct when the meta-analysis effect size was positive and had a 95% CI which did not cross zero. Forest plots were also generated for pooled data. Unpooled data were presented in table format, allowing comparisons between each outcome. Analyses were performed in R (38) using the metafor package (35). UR and VS contributed to and confirmed the synthesis of the extracted data. Discrepancies in data synthesis were discussed amongst the authors until consensus were reached, with NK and NS serving as arbitrators.

With respect to the secondary aim, given the diversity of intervention features, context, and population, it was not possible to make a direct link between intervention features and outcome. Therefore, key behavioral intervention features of included studies were recorded and tabulated.

## Results

### Selection of Studies

**Figure 1:**
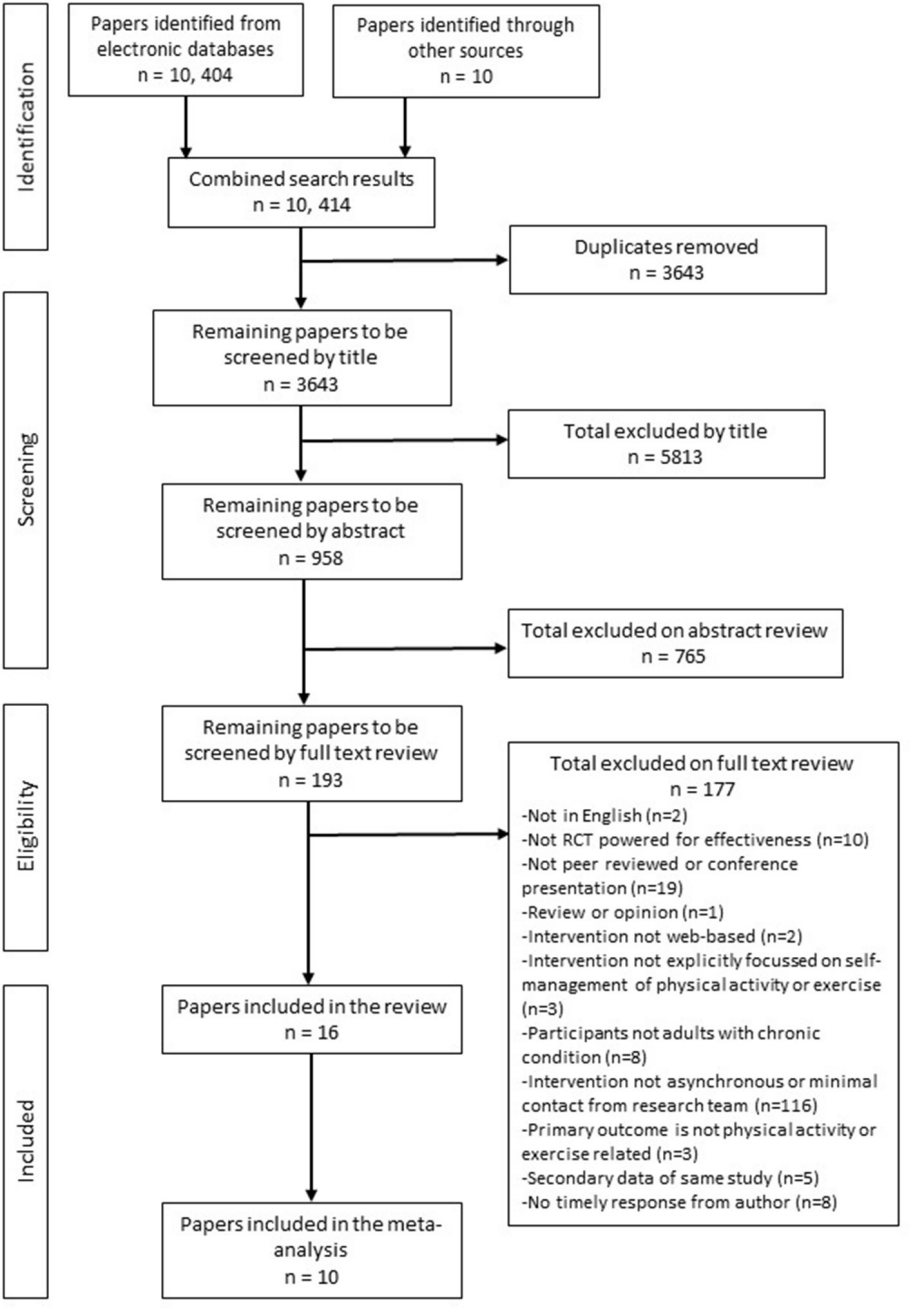
Flow chart summarizing the study selection process.

A flow diagram of the identification, screening and selection of papers is presented in Figure 1. There was 98% agreement with the co-author (NS) during the screening phase, discrepancies were resolved by discussion. Following the full text review, 177 papers were excluded because they did not meet the a priori criteria (see Supplementary File 1 for a table of excluded papers). Three papers required discussion with arbitrator (NK). Sixteen papers met the criteria and were included in the review with ten included in the meta-analysis.

### Risk of Bias

**Figure 2:**
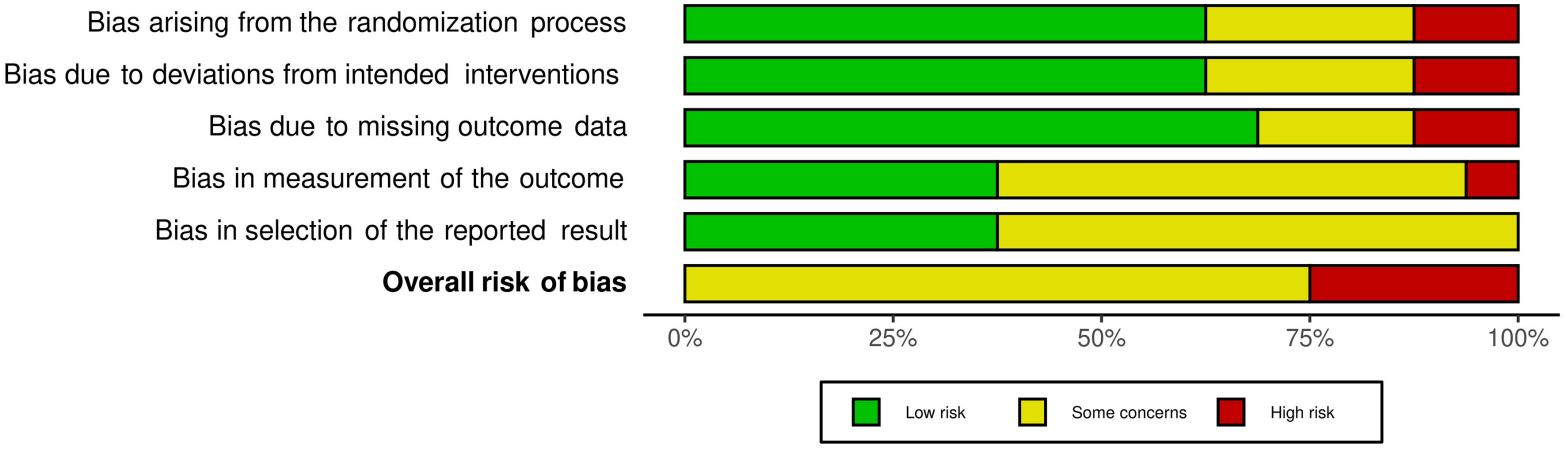
Weighted summary plot of the overall type of bias encountered in the included studies.

The risk of bias assessment for included studies is available in Supplementary File 2. Agreement between reviewers was high (92%). Consensus was reached where there was discrepancy. Overall, no studies were assessed as having a low risk of bias. The majority were assessed as having some risk of bias (39–48), with the remaining five judged to be at high risk of bias in at least one domain (49–53). As shown in the weighted summary plot (Figure 2), this could be attributed to having potential bias in the selection of reported results. Many studies did not have a published protocol, making it difficult to determine if analyses were carried out according to a pre-specified plan. Studies were also at risk of bias due to outcomes of interest being self-reported by participants who were unblinded to intervention status. Missing outcome data (for example, participants lost to follow up) also contributed to the bias.

### Characteristics of Selected Studies

Included papers were published between 2013 and December 2021 (end of collection period). Publications in this research area have been increasing recently with half published in the last 2 years (40, 44–46, 51–54). Studies were conducted in Germany (41, 44), the Netherlands (39, 45), the United Kingdom (49, 54), Korea (42, 52), Australia (40), Brazil (48), Canada (53), China (47), Hong Kong (46), New Zealand (43), Norway (50), and the United States (51).

Interventions were compared to usual care or wait list groups in six studies (39, 41, 43, 46, 49, 54), to paper based interventions in four studies (42, 44, 47, 48), to another form of online intervention in three studies (40, 52, 53) and to a supervised intervention (50). One study used the intervention of interest (minimal contact self-guided intervention) as the comparison group when testing versions of online and blended interventions (51). Table 3 contains characteristics of the included studies.

**Table 3:** Characteristics of the randomized controlled trials, participants, and interventions.

**References, country**	**Inclusion criteria**	**Sample size (*n*) mean age (yr) females (F) males (M)**	**Intervention details and use of theory**	**Comparison**	**Outcomes of interest**	**Intervention duration (D) Frequency of delivery (F) Intended frequency of exercise (E)**
Bossen et al. (39) Netherlands	**Self-report OA knee/hip**, 50–75 yrs, self-report inactivity, no treatment in last 6 months, no contra-indications to exercise internet access	*n* = 199 Age = 62 yrs F = 129 M = 70	Eight weekly modules of Behavioral Graded Activity (Join 2 move) of participant's identified favorite activity. Automatically increased depending on answers from pain and performance scale. Intervention based on operant behavior principles with aim of increasing PA.	Wait list	PA (self-reported) *via* PASE PA (instrumented) *via* ActiGraph GT3X accelerometer KOOS/HOOS Self-perceived effect	D = 9 weeks F = 1 x week E = determined by users
Chapman et al. (40) Australia	**Breast cancer** survivors, ≥18 yrs, completed treatment, no contra-indications to exercise	*n* = 101 Age = 59.1 yrs F = 101	Online volitional help sheet (VHS) which presents most likely barriers to PA. Participants prompted to select possible coping strategies to be more equipped if situation arises. Intervention based on TTM and implementation-intention with aim of increasing PA.	Online standard implementation intention version that presents most likely PA barrier scenarios but requires participant to self-generate possible coping strategies.	PA (self-reported) *via* GSLTPAS to determine moderate PA: LSI-MSPA	Once off intervention E = not applicable
Crooks et al. (49) UK	**COPD**, 40–80 yrs, FEV_1_ >50% and FEV_1_/FVC <70% predicted or COPD diagnosis within 12 months, current or ex-smoker, internet access, able to use web platform in English	*n* = 60 Age = 66.1 yrs F = 29 M = 31	myCOPD online application and tile platform covering variety of self-management topics. Users able to input data for more tailored advice. Intervention not based on theory. Aims to improve areas of body structure and function and activity.	Usual COPD management for study duration. After study completion, offered app access.	CAT 7-day step count *via* Fitbit at baseline and at completion	D = 12 weeks F = variable, some expected daily (like exercise related content) others every few weeks E = unclear, likely daily
Geraghty et al. (54) United Kingdom	**Dizziness** lasting 2 yrs made worse with head movement, ≥50 yrs, access to internet	*n* = 296 Age = 67.4 yrs F = 197 M = 99	Six-week balance retraining, rehabilitation, adaptation, and habituation program on improving symptoms. Includes head movements to promote reduction of movement provoking dizziness and reduce avoidance behaviors. Intervention based on SRT, CBT.	Standard non-web-based care consisting of reassurance, symptomatic relief with or without educational information.	Symptom severity *via* VSS-SF	D = 6 weeks F = 1 × week E = 2 × day ×10 min
Haglo et al. (50) Norway	**Inflammatory rheumatic diseases**, ≥18 yrs, diagnosed with either RA, SpA, SLE, not familiar with HIIT	*n* = 40 Age = 49 yrs F = 23 M = 17	Myworkout GO smartphone app to guide and deliver 4 ×4 min HIIT at a % of HR max. App provides display of progression and estimation of work performed automated scheduled next exercise time. Intervention not based on behavioral theory. Aims to improve areas of body structure and function and activity.	Supervised 4 ×4 HIIT	VO_2_ max *via* Metamax II	D = 10 weeks F = 2 × week E = 2 × week
Holtdirk et al. (41) Germany	**Breast cancer** survivors, 30–70 yrs, diagnosis <5 yrs ago, completed acute treatment >1 month prior to study entry with discharge letter proof, able to speak and read German	*n* = 363 Age = 49.9 yrs	Sixteen multimodal web-based modules that registered users can select and work through. Subsequent content is continuously tailored based on user response. Daily text messages remind and motivate users to use the program. Intervention is based on CBT.	Usual care and wait list of 3 months for access to the intervention.	PA (self-reported) *via* IPAQ	D = 12 weeks F and E= self-paced
Kelechi et al. (51) United States	**Venous leg ulcers**, ≥18 yrs, impaired mobility resulting in inability to walk 100 feet without resting, no current PA, requiring wound care, no arterial insufficiency, able to don the slipper to which BEAT accelero-meter is affixed	*n* = 24 Age = 64.9 yrs F = 14 M = 10	COMPARISON Six-week progressive exercise intervention delivered through app (FOOTFIT). Non-exertional exercises (Conditioning physical Activities for lower Leg Function-CALF) tracked with Bluetooth Enabled triaxial Accelerometer Tracking (BEAT). Intervention not based on theory. Aims to improve areas of body structure and function and activity.	INTERVEN-TION FOOTFIT+ is FOOTFIT, CALF and BEAT with added phone, email, or text messaging connectivity to wound care providers. Intervention not based on theory. Aims to improve areas of body structure and function and activity.	ROM of ankle Strength of ankle FAAM 6 MWT	D = 6 weeks F = all at once E = 3 × day × 15 s progressed every 2 weeks
Kwon et al. (52) Korea	**COPD**, ≥20 yrs, FEV <80%, >150 m in 6 MWT, Android smart-phone user	*n* = 85 Age = 64.3 yrs F = 15 M = 70	efil breath fixed-interactive app uses participant data from baseline and current exertion level feedback to tailor walking prescription. Intervention not based on theory. Aims to improve areas of body structure and function and activity.	efil breath fixed app uses pre-determined walking distances and progresses when participant achieves certain targets and usual care with no app.	6 MWT CAT mMRC	D = 12 weeks F = daily E = 7 × week × 30 min
Lee et al. (42) Korea	**Breast cancer** survivors, hemoglobin over 10 g/dl, not meeting exercise or healthy eating goals, access to computer, home internet, mobile phone user	*n* = 57 Age = 42.4 yrs F = 57	Health Planner 5 portions: assessment that leads to tailored plan for each participant: education (tailored info provision), action planning (goal setting, scheduling, keeping a diary), automatic (tailored) feedback. Intervention based on TTM with aim of increasing PA.	50-page educational booklet on exercise and diet.	PA (self-reported) *via* 7-day exercise diary (minutes per week ≥4 METs)	D = 12 weeks F = 2 × week E = 5 × week × 30 min
Liu et al. (53) Canada	**HTN** (stage 1 or 2), 35–74 yrs, stable meds before enrolment, if on antihypertensives: SBP 130 and DBP 85	*n* = 128 Age = 56.9 yrs F = 61 M = 67	Automated e-counseling: Participant identifies areas to address and is provided with pre-determined expert driven suggestions which are informed by foundation questionnaire. Intervention based on TTM with aim of increasing PA.	Automated e-counseling: Participant identifies areas to address but is self-reliant (user-driven) for suggestions and Control group	SBP PA (instrumented) *via* daily step count *via* XL-18CN pedometer	D = 16 weeks F = 1 × week E = 5 × week × 30 min
Maddison et al. (43) New Zealand	**IHD**, ≥18 years, clinically stable, able to perform exercise, able to understand and write English, access to Internet.	*n* = 171 Age = 60.2 yrs F = 32 M = 139	Personalized, automated package of text messages *via* mobile phones aimed at increasing exercise behavior. Intervention based on SET with aim of increasing PA.	Usual care	Peak VO_2_ *via* Moxus PA *via* IPAQ	D = 24 weeks F = 6 × week E = 5 × week × 30 min
Nasseri et al. (44) Germany	Progressive **MS**, 18–60 yrs, EDSS below 6.5	*n* = 38 Age = 51.1 yrs F = 19 M = 19	12-week app-based information package on exercise including text, figures, videos and accelerometery activity feedback. Intervention not based on theory. Aims to improve areas of body structure and function and activity.	Paper based leaflet with information on generalized exercise.	6MWT 5xSTS PA (self-reported) *via* GSLTPAS to determine moderate PA: LSI-MSPA PA (instrumented) *via* Actigraph accelerometer	D = 12 weeks F = not specified but assumed delivered all at one time E = not specified but assumed daily
Van Vugt et al. (45) Netherlands	**Dizziness** ≥1 month and ↑ with head movement, ≥ 50 yrs, Dutch speaker, access to internet and email	*n* = 322 Age = 67 yrs F = 197 M = 125	Six-week stand-alone online vestibular rehab (VR)-adaptation, habituation with relaxing, cognitive restructuring, engagement features. Intervention based on CBT, SET, exposure-based behavior.	Usual care with no VR	Symptom severity *via* VSS-SF	D = 6 weeks F = 1 × week E = 2 × day × 10 min
Wong et al. (46) Hong Kong	**CHD**, ethnic Chinese 45–65 yrs, regular treatment for CHD, able to use internet at home	*n* = 438 Age = 52.3 yrs F = 149 M = 289	eHES website representing constructs such as “cues to action” and “enhancing self-efficacy” that allow self-monitoring of individual health and exercise. Intervention based on HBM.	Usual care including routine medical visits and a paper based educational leaflet.	PA (self-reported) *via* GSLTPAS. Self-efficacy for exercise	D = 24 weeks F = not specified but assumed delivered all at once E = 5 × week × 30 min
Wong et al. (47) China	**Metabolic Syndrome**,>50 yrs, ethnic Chinese, had MetS as defined by waist circumference, triglycerides, HDL, BP and fasting plasma glucose measures, own smartphone access	*n* = 77 Age = 58 yrs F = 43 M = 43	MetS app to support_initiation and maintenance of healthy behaviors relating to monitoring weight, diet, and exercise. Intervention based on HBM.	Booklet providing MetS management information.	PA (self-reported) *via* GSLTPAS. Self-efficacy for exercise	D = 12 weeks F = not reported E = 5 × week × 30 min
Yuan et al. (48) Brazil	**Fibromyalgia**, 19–59 yrs, diagnosis by American College Rheumatology diagnostic criteria, smartphone user, completed elementary school education	*n* = 40 Age = 43 F = 39 M = 1	ProFibro App providing self-management through animation, self-monitoring, family adjustment, sleep hygiene scheduling, graded exercise, hints through notifications. Intervention not based on theory. Aims to improve areas of body structure and function and activity.	64-page booklet to replicate app.	Pain *via* WPI. Pain *via* VAS. Symptom severity *via* SS	D = 6 weeks F = not reported E = not reported

*2 MWT 2 min walk test, 4 MET moderate aerobic exercise that consumed at least 3.5 ml/O_2_/kg/min or ≥150 min/week; 5 STS five time sit to stand; 6 MWT 6 min walk test, app application; AROM, active range of motion; BP, blood pressure; CAT, COPD assessment test; CBT, cognitive behavioral theory; CHD, Coronary heart disease; COPD, chronic obstructive pulmonary disease; DASH, Disabilities of the Arm; Shoulder and Hand; EDSS, Extended Disability Severity Scale, ext extension; FAAM, Foot and Ankle Mobility Measure; g/dl grams per decalitre; GP, general practitioner; GSLTPA, Godin-Shephard Leisure-Time Physical Activity Score; HBM, health belief model; HOOS, Hip Osteoarthritis Outcome Score; HTN, hypertension; IHD, ischemic heart disease; IPAQ-LF, International Physical Activity Questionnaire-Long Form; Kg Kilogram; KOOS, Knee Osteoarthritis Outcome Score; LSI-MSPA, Leisure Score Index of moderate-strenuous exercise; m meters; MET, metabolic equivalent; mMRC, Modified Medical Research Council Dyspnea Scale; MS, Multiple Sclerosis; OA, osteoarthritis; op operative; OT, occupational therapy; PA, physical activity; PASE, Physical Activity Scale for the Elderly; prn as deemed appropriate; RCT, randomized controlled trial; ROM, range of motion; SBP, systolic blood pressure; SET, self-efficacy theory; SRT, self-regulatory theory; TTM, transtheoretical model; VO_2_, maximum rate of oxygen consumption; VR, virtual reality; VSS-SF, vestibular symptom scale-short form, yrs years*.

### Characteristics of Participants

Participants were recruited from primary health care, community settings and online databases. The number of participants within studies ranged from 24 to 438, with a combined total of 2,439 participants across studies (median *n* = 93). The average age of participants across studies was 57.1 years with the mean age range from 43 to 67 years. Participants presented with the following chronic conditions: breast cancer (40, 42, 50), dizziness and vestibular syndrome (54, 55), heart disease (43, 46), chronic obstructive pulmonary disease (49, 52), fibromyalgia (48), hypertension (53), inflammatory rheumatic diseases (50), metabolic syndrome (47), progressive multiple sclerosis (44), osteoarthritis (39), and venous leg ulcers (51). Table 3 provides details for all included studies.

### Outcome Measures

Outcomes of interest were those related to the PA or exercise interventions that measured an activity or body function (24). These were grouped into change in PA that was self-reported and change in PA that was measured by instrumentation. Changes in body functions and symptoms were also reported. Change in PA was meta-analyzed in ten of the included studies. This was measured using self-reported questionnaires (39–44, 46, 47) and instrumented devices (accelerometers, pedometers or wearable devices) (39, 44, 49, 53). The remaining relevant outcomes varied considerably among studies. Therefore, they were broadly grouped, as per the ICF framework (24), into change in body functions and symptoms and included measures such as walking endurance, measured by the 6-min walk test (44, 51, 52), strength (44, 51), vestibular symptoms (54, 55), peak oxygen uptake (43, 50), pain (48), foot and ankle mobility (51), dyspnea (49, 52), and range of motion (48, 49, 51–53). Self-reports of perceived effect and self-efficacy of exercise were also measured (39, 46, 47). Bossen et al. (39) and Kwon et al. (52) used the Knee Osteoarthritis Outcome Score and Hip Osteoarthritis Outcome Score. These measure symptoms, activities of daily living and quality of life; the scoring of each outcome precludes the ability to report these constructs separately.

### Characteristics of the Interventions

#### Modes of Delivery

There were a variety of modes of delivery and technologies used across interventions. Eleven of the interventions involved computers (39–43, 46, 49, 52–55). Mobile or smartphones were used in nine studies (41–44, 47, 48, 50–52). Almost half the studies were testing a website specific to the study (39, 41–43, 46, 54, 55) while three used existing platforms (40, 52, 53). Applications specifically designed for the study were used in seven studies and involved a smartphone or computer (44, 47–52). Communication with participants was carried out via automated email (39, 46, 49, 53–55), automated SMS text messaging (41–43) or the study app features (47, 48, 50–52). Biosensors were used in five studies (39, 43, 49, 51, 52).

#### Parameters of Intervention Delivery

Table 3 provides details on key intervention parameters. Intervention duration ranged from a one-off session to 24 weeks. The expected frequency of the exercise or PA&E performance varied. This ranged from a one-off instructional session (40) to asking participants to participate repeatedly throughout the day (51, 54, 55), once daily (44, 49, 52), several times a week (42, 43, 46, 50, 53), or as determined by the participant (39, 41, 52).

#### Theoretical Basis

Just over half of the included studies reported using theory to inform their interventions, with three referencing more than one theory. The transtheoretical model was the most frequently cited (40, 42, 53). Self-regulatory theory, cognitive behavioral theory, social cognitive theory, exposure-based behavior principles, implementation-intention-based principles, health belief model and operant behavior principles were also each used once (39–41, 43, 46, 47, 54, 55). No underpinning theory was reported for six of the self-guided interventions (44, 48–52).

#### Behavioral Intervention Strategies

The interventions used a combination of behavioral strategies and features to support PA&E behavior (see Table 4). A commonly used feature was instruction on how to perform the PA or exercise (*n* = 14). Variations included generalized automated PA or exercise information, tailored exercise provision, and tracking and recording of PA or exercise performed. The use of feedback and monitoring (*n* = 13) was also used in most interventions. Other strategies involved goal setting (*n* = 11) and the use of prompting features (*n* = 10). Fewer than half the interventions incorporated action and coping plans (*n* = 5), online resources of supplemental PA or exercise information (*n* = 4) or testimonials or case studies (*n* = 5). None of the included studies used gamification approaches. Most interventions employed several strategies concurrently.

**Table 4:** Behavioral intervention features and strategies used.

**References, Country**	**Instruction on how to perform PA or exercise**	**Use of feedback and monitoring in PA or exercise**	**Use of goal setting for PA or exercise**	**Use of prompts for PA or exercising**	**Use of action and coping plans for PA or exercise**	**Use of online resources for additional PA or exercising**	**Use of testimonials in benefits of PA or exercise**	**Use of gamification to encourage PA or exercise**
Bossen et al. (39) Netherlands	✓	✓	✓	✓		✓		
Chapman et al. (40) Australia			✓		✓			
Crooks et al. (49) UK						✓		
Geraghty et al. (54) United Kingdom	✓	✓	✓				✓	
Haglo et al. (50) Norway	✓	✓	✓	✓				
Holtdirk et al. (41) Germany	✓	✓	✓	✓	✓		✓	
Kelechi et al. (51) United States	✓	✓		✓				
Kwon et al. (52) Korea	✓	✓	✓					
Lee et al. (42) Korea	✓	✓	✓	✓	✓			
Liu et al. (53) Canada	✓	✓	✓	✓	✓			
Maddison et al. (43) New Zealand	✓	✓	✓	✓	✓	✓	✓	
Nasseri et al. (44) Germany	✓	✓					✓	
Van Vugt et al. (45) Netherlands	✓		✓	✓		✓	✓	
Wong et al. (46) Hong Kong	✓	✓		✓				
Wong et al. (47) China	✓	✓	✓					
Yuan et al. (48) Brazil	✓	✓		✓				

### Effectiveness of Interventions

Effectiveness was measured by change in PA&E and change in body function and symptoms. These were defined and measured in a variety of ways.

#### Physical Activity

**Figure 3:**
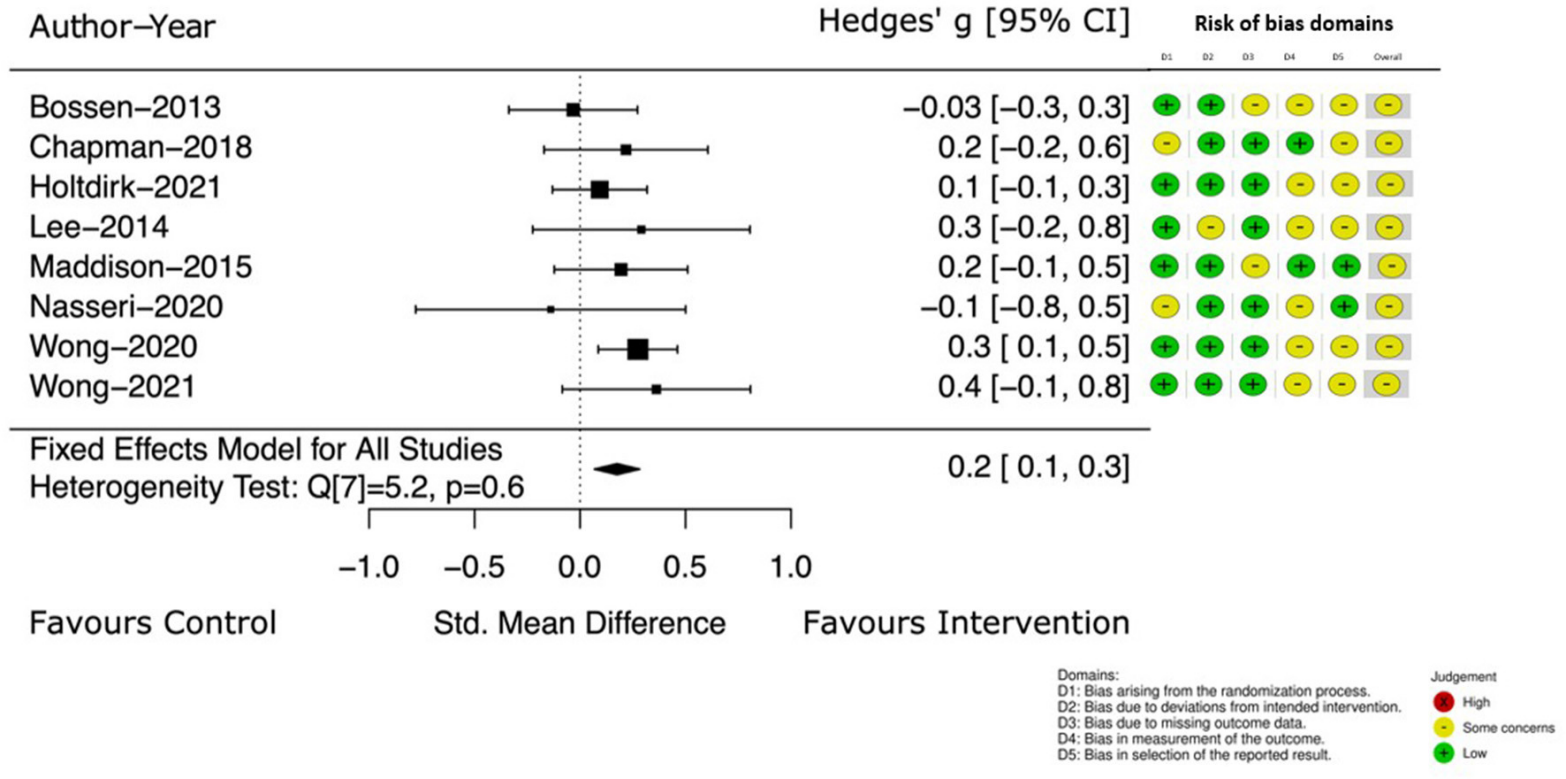
Self-reported PA at end of intervention.

Change in self-reported PA was the outcome of interest in eight of the included studies (39, 41–44, 46, 47). Figure 3 details the Hedge's g between group difference, with 95% CI, at the earlier assessment point, taken at the end of intervention (which ranged from 4 to 24 weeks). The Hedge's g between groups favored the intervention group with a small, estimated effect of 0.2 [95% CI (0.1, 0.3)].

**Figure 4:**
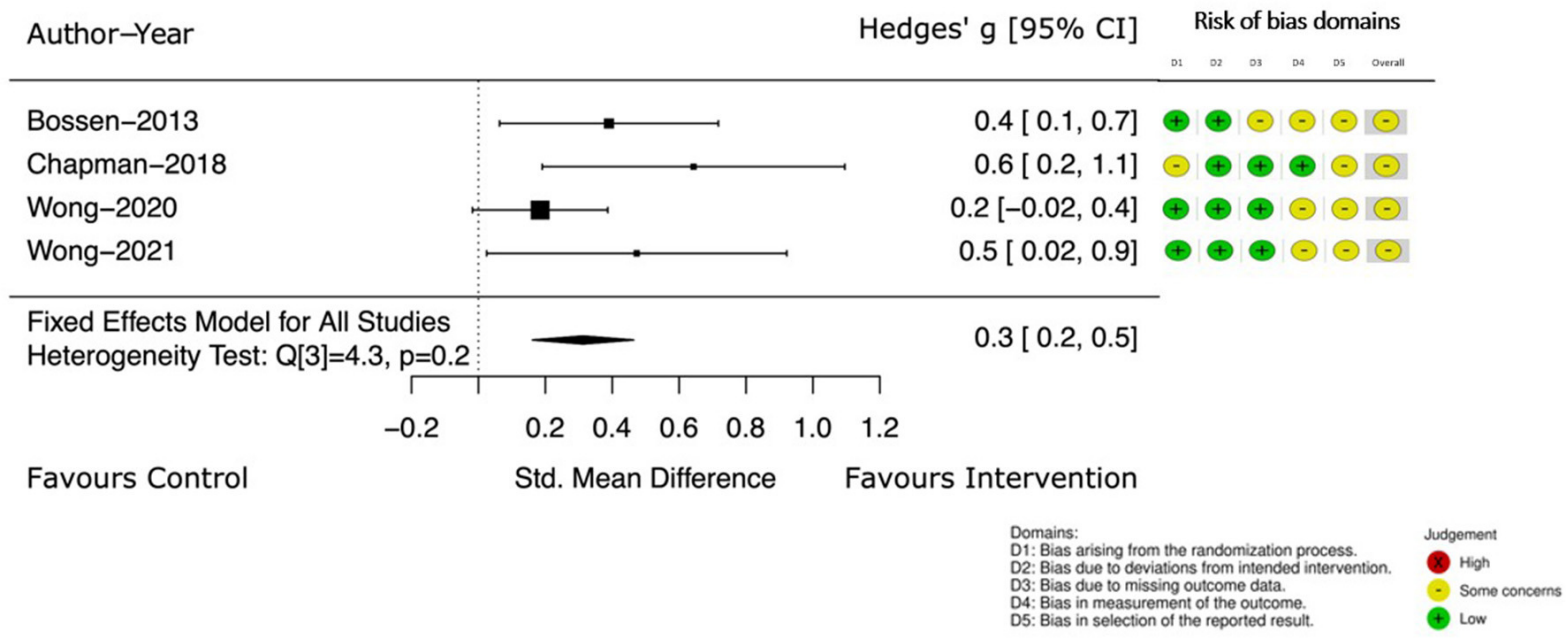
Self-reported PA at follow up.

Four of these studies (39, 40, 46, 47) continued to measure self-reported PA at a follow up point (12–52 weeks from the end of intervention). Pooled results (39, 40, 46, 47), demonstrate the effect of the intervention. The Hedge's g between groups continued to favor the intervention with an increased estimated effect [Hedge's g 0.3, 95% CI (0.2, 0.5)] (see Figure 4).

**Figure 5:**
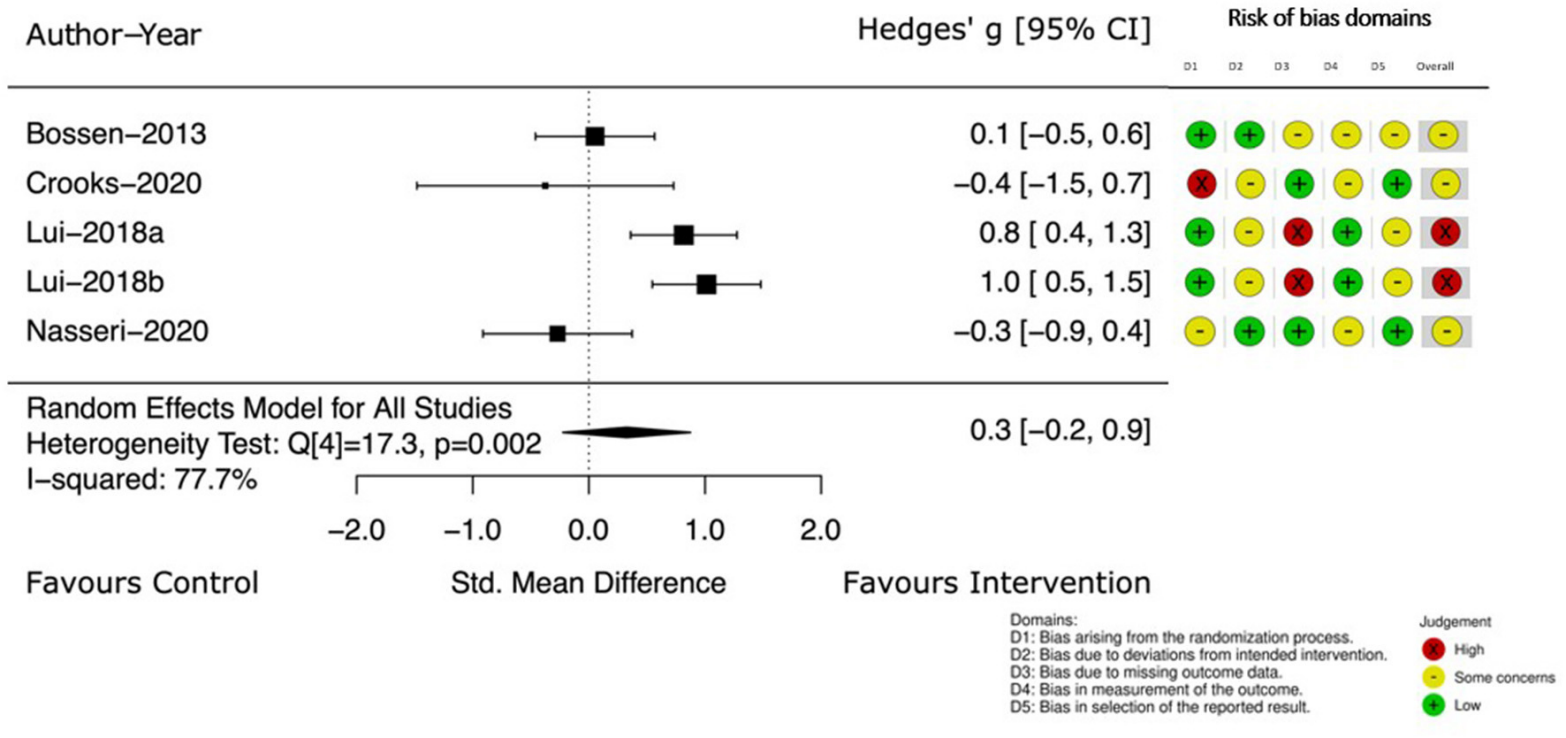
Instrumented measurement of PA at end of intervention.

Change in PA, measured by instruments such as accelerometers or pedometers was described in four studies (39, 44, 49, 53). The Hedge's g between groups and 95% CI at the completion of the intervention are displayed in Figure 5. The small treatment effect on PA was in favor of the intervention group (Hedge's g 0.3) but the CI crossed the no effect line [95% CI (−0.2, 0.9)] and heterogeneity was 77.7%.

#### Body Functions and Symptoms

Meta-analysis was not conducted on outcomes related to body functions and symptoms due to the high heterogeneity of measures employed. There was variation in the size of effect and statistical significance across the studies. Refer to Supplementary File 3 for details of each study, the extracted outcome measures, assessment points, mean group differences and estimated effects. Despite studies showing some risk of bias, there are some results that were of note. Effects (Hedge's g 0.3–0.8) were reported in two separate studies evaluating 6-week vestibular interventions at the 12- and 24-week post intervention time points (54, 55). Both interventions had a theoretical basis and incorporated several behavioral strategies. There was also evidence of a small positive effect of graded-activity on physical function at 12 weeks [Hedge's g 0.4, 95% CI (0.1, 0.7)] but not at the 52-week follow up in a 9-week PA intervention for people with knee and hip OA (39). Self-perceived effect and exercise self-efficacy were measured in two studies (39, 47). A moderate effect [Hedge's g 0.6, 95% CI (0.3, 0.9)] was noted for self-perceived effect after the 9-week PA intervention. Similarly, a moderate effect was seen in self-efficacy for exercise [Hedge's g 0.5, 95% CI (0.1, 1)] at the end of a 4-week intervention and maintained [Hedge's g 0.6, 95% CI (0.2, 1.2)] at the 12 week follow up. Theoretical underpinnings and behavioral features were also incorporated in these interventions. Studies that did not have a theoretical basis did not report any effects.

When looking across study exercise parameters, there was a large variation in dosage (amount x frequency x duration) of prescribed exercise. There did not appear to be a clear relationship between dosage and effect.

## Discussion

### Principle Findings

This is the first systematic review to conduct a meta-analysis of outcomes following self-guided digital PA&E interventions in people living with chronic conditions. This work advances the evidence in digital interventions for PA&E. The more stringent inclusion criteria for minimal contact used in this review meant that none of the studies in the review by Bossen and colleagues (18) met the threshold to be included.

The findings of our review indicate a positive effect of self-guided digital interventions on PA that were seen at the end of intervention and sustained at follow up, for people living with chronic health conditions. These findings differ from the findings of Bossen and colleagues of conflicting evidence on the effectiveness of web-based PA interventions in this population (18). Our findings reflect the developments in technology that have enabled persuasive and engaging elements to be embedded into digital interventions (56, 57). These may help overcome some of problems of low uptake and adherence (58).

There was a small but significant effect seen with self-reported PA at the end of interventions. Direito and colleagues (9), investigating the effect of mHealth technologies on self-reported PA in healthy participants, found a comparable effect that was not significant. Kwan et al.'s (59) review of eHealth interventions promoting PA in older adults found that self-report PA was significantly increased compared with the control groups. However, since the interventions in both reviews included contact and personal consultation, a cautious approach to comparison is needed.

Objectively measured change in PA in the current review showed a small effect that was not significant. This has also been reported by others (9, 60) who have found insignificant effects favoring mobile and app-based interventions that were not strictly self-guided. There is an assumption that objective PA measures more accurately reflect actual changes in PA. However, Kayes and McPherson (61) argue measurement tools such as accelerometers and pedometers have not always been validated in people living with chronic health conditions. Despite significant advances in the objective measurement of PA in these populations, questions remain regarding the validity and reliability of these devices in some groups, such as people with slow walking speeds or those with higher levels of disability (62, 63).

Our findings of sustained PA improvement beyond the intervention, is in contrast with previous work. In-person PA interventions have been shown to increase PA levels in the short to medium term (7 weeks to 1 year) but not in the long term (at or over 1 year), in community dwelling adults with or without a long-term health condition (64, 65). Our findings are supported by Davies et al. (8) who conducted a review of web-based PA interventions and found an overall small but significant mean effect of sustained PA when looking at a follow up of at least 6 months after the intervention. Regardless of the length of follow up, the persistence of change seen, despite the removal of intervention, is encouraging.

The prolonged effect observed in some studies may be linked to the use of behavioral strategies in the interventions. The most common intervention strategies and features used were instructions on how to perform the PA or exercise, goal setting, and the use of feedback and monitoring. These align with the behavior change techniques (BCT) taxonomy clusters proposed by Michie and colleagues (27). The self-guided interventions with the larger effect sizes employed strategies from at least three of these clusters (39, 42, 46, 53–55). This finding is supported by research showing that the use of techniques from three BCT clusters are needed to produce effects on PA in face-to-face interventions (66, 67). For self-guided digital interventions, employing self-regulatory techniques may be the most effective way to support PA engagement (28, 29, 68). Michie et al. (68) found that interventions combining self-monitoring (using feedback) in combination with other features that encouraged self-regulation (goal setting, action and coping plans) were more likely to lead to intervention effectiveness (69). These strategies can influence behavior *via* mechanisms such as problem-solving, promoting self-efficacy or diminishing the impact of barriers to behavior change (70). They may also address the intention behavior gap (71). In this review, fewer than half the interventions reviewed used action and coping plans or other strategies that promote self-regulation. The lack of theoretical underpinning found in many of the studies may help explain their choice of intervention features and non-significant results. While we did not explicitly set out to determine the effectiveness of behavioral features, interventions that were effective did include behavioral features. Determining which specific features might best be implemented to support sustained use and engagement with self-guided interventions would be a future research direction.

The lack of an apparent relationship between exercise dose and effect suggests that the prescribed dose may be only one factor of an intervention that influences participant outcomes. The behavioral strategies embedded in the interventions influence exercise completion and, therefore, are an important factor in the reported interventions. The lack of reporting of exercise completion makes it difficult to appreciate how these factors contribute to outcome.

### Strengths and Limitations

This review focused on investigating the effect of digital self-guided interventions on PA&E and fills a gap in the literature in this growing area. We included all forms of digital interventions that could be delivered remotely, without ongoing human interaction. The comprehensive search, using several databases, identified studies previously not included in similar reviews, and the broad range of outcomes make the findings generalizable to a wider range of populations, interventions, and environments. We identified behavioral strategies using well-established frameworks (27) that allows for transparency and a clear understanding of intervention features that may help to explain reported effects. This review has also reported metrics for discrete end of intervention and follow up outcomes and meta-analyses for PA outcomes for self-guided digital PA interventions for people living with chronic conditions.

People living with chronic conditions face additional challenges in undertaking PA&E compared to a healthy population. Acknowledgment of the inherent heterogeneity that comes with each health conditions should sit alongside any the generalization of these findings, particularly when a significant portion of the people included in this review were living with cardiorespiratory, neurological or oncology conditions and were under the age of 60. Despite this, there is much we can learn by looking across populations, particularly when those populations share characteristics beyond diagnosis such as chronicity and complex barriers to engagement with PA&E.

To be included in the review, papers needed to have been peer reviewed to address the question of effectiveness, which may have introduced publication bias. Other criteria were that interactions with the intervention had to be automatically generated. Screening of the papers for this criterion was not straightforward due to the lack of a standard definition of what constitutes minimal contact. We selected papers based on our operational definition and consequently many interesting and valuable digital PA&E interventions were excluded. For included papers, data were frequently incompletely or inconsistently reported, and some were analyzed as intention to treat while others were not. This made analysis of the results difficult and necessitated computation using reported data. The wide variation in health-related body function and symptoms outcomes prevented us from pooling this data. Therefore, we synthesized the results in a way that allowed the magnitude and range of effects to be appreciated.

The studies included in this review demonstrated some or high risk of bias and so findings should be interpreted with caution. The nature of the minimal contact digital intervention creates increased opportunities for bias with attrition, and the reporting of outcomes remotely.

### Future Recommendations

Future work should continue to investigate which intervention features and exercise parameters lead to the best effect. Effort should continue to ensure complete reporting of the intervention, the behavioral interventions used, and treatment fidelity, including recording the participant's completion of the prescribed dose. Authors also need to ensure that adverse events are explicitly sought and consistently reported. This was not the case in many of the studies included in the current review. For interventions that are self-directed and have minimal contact with health professionals, understanding intervention safety is an important component of the trialing phase.

The proportion of participants lost to follow up in the studies in this review demonstrates that maintaining engagement with low contact digital interventions is challenging (72, 73). Given the added barriers experienced by these people (74), tailoring the intervention to the individual needs to be considered and addressed (75).

Researchers are encouraged to broaden the scope of populations involved in this type of research. For the current review, the population of interest focused on people living with chronic conditions that included a range of non-communicable diseases. Of note was the lack of studies involving people living with disabilities, a large group who also would benefit from self-guided and digital interventions. Finally, the participant's voice is notably absent from much of this research.

Our research group is currently exploring many of these areas. For example, we are applying an interpretive descriptive study design to explore what makes a self-guided digital intervention more acceptable to users, with the aim of developing interventions which increase uptake and engagement (76). The findings from this current review and the interpretive descriptive study will inform our development a self-guided digital intervention to help treat shoulder pain for people living with spinal cord injury (76).

## Conclusion

This review found a positive effect in favor of self-guided digital PA&E interventions on PA outcomes and a selected number of body functions and impairments at the end of intervention and at follow up, in people living with chronic conditions. Interventions that employ behavioral strategies, underpinned by a theoretical framework, have the potential to support self-regulation and sustained PA at least as well as interventions with supervision.

## Data Availability Statement

The original contributions presented in the study are included in the article/Supplementary Material, further inquiries can be directed to the corresponding author.

## Author Contributions

VS, NK, and NS contributed to conception, design of the study, and analyzed intervention features. VS performed the initial search and organized the database. VS and NS reviewed study selection. VS and NK analyzed risk of bias of selected studies. UR provided statistical support. All authors read and approved the final manuscript.

## Conflict of Interest

The authors declare that the research was conducted in the absence of any commercial or financial relationships that could be construed as a potential conflict of interest.

## Publisher's Note

All claims expressed in this article are solely those of the authors and do not necessarily represent those of their affiliated organizations, or those of the publisher, the editors and the reviewers. Any product that may be evaluated in this article, or claim that may be made by its manufacturer, is not guaranteed or endorsed by the publisher.
