# Supplementary material for: The Effectiveness of Self-Guided Digital Interventions to Improve Physical Activity and Exercise Outcomes for People With Chronic Conditions: A Systematic Review and Meta-Analysis
Source: Front Rehabil Sci. 2022 Jun 24;3:925620. doi: 10.3389/fresc.2022.925620 (PMC9397696; doi:10.3389/fresc.2022.925620)
Supplement: Supplementary file 1 [file Data_Sheet_1.docx]

**Supplementary File 1.** Table of excluded randomised controlled trials

| Author and date | Population | Intervention | Criteria used to exclude | Reason for exclusion |
| --- | --- | --- | --- | --- |
| Abadiyan et al., 2021 | Non-specific neck pain | Smartphone app | Intervention more than just initial contact-ongoing intervention contact not generated automatically | The participants in each experimental group  performed the intervention in a clinic and were supervised by physiotherapists and two corrective exercise trainers specialized at postural reeducation exercises by  the physiotherapists. |
| Allen et al., 2018 | Knee osteoarthritis | Internet-based exercise training vs face-to-face physiotherapy vs wait list | Intervention more than just initial contact-ongoing intervention contact not generated automatically | Participants could also communicate with study personnel via the internet program and request a reply if needed. Any questions pertaining to exercises or symptoms were handled by a healthcare professional associated with the study (i.e., physician or physical therapist) |
| Allen et al., 2021 | Knee osteoarthritis | Progressively more supported exercise intervention for physical activity and exercise related to knee OA | Intervention more than just initial contact-ongoing intervention contact not generated automatically | Intervention did not compare minimal contact to control group. Intervention involved a combination of contact, depending on participant criteria |
| An et al., 2021 | Adhesive capsulitis | Deep learning-based smartphone application vs CPM | Intervention was not explicitly aimed at self-management of physical activity or exercise | Single session intervention comparing two intervention techniques. |
| An et al., 2021 | Knee osteoarthritis awaiting total knee arthroplasty | Preoperative telerehabilitation (PT) | Intervention more than just initial contact-ongoing intervention contact not generated automatically | All interventions were performed at home using a smartphone or tablet via a two-way video call. The therapist provided supervision and intervention with real-time visual feedback and verbal cues. |
| Anan et al., 2021 | Neck/Shoulder Pain/Stiffness | Artificial intelligence-assisted health program | Participants are not adults with a chronic condition | Not clear on if participants had acute or chronic conditions |
| Antypass et al., 2014 | Cardiovascular disease | Tailored internet and mobile based intervention for PA vs basic internet intervention with general info | Intervention more than just initial contact-ongoing intervention contact not generated automatically | Discussion groups with moderation by research team/health professional.  Participants could seek assistance by calling a physiotherapist during working hours. |
| Arbour-Nicitopolous et al., 2017 | Spinal cord injury | SCI GetFit toolkit vs online Physical Activity Guidelines-SCI | Primary outcome is not physical activity or exercise related as per the ICF Framework | Outcomes include intentions to engage in PA; self-efficacy; outcome expectancies; action planning. |
| Asano et al., 2021 | Stroke | Singapore Tele-technology Aided Rehabilitation in Stroke (STARS) trial | Intervention more than just initial contact-ongoing intervention contact not generated automatically | Sessions involve Facetime video calls during a scheduled tele-consultation. Therapists also enter clinical notes and parameters into the application. |
| Asklund et al., 2015 | Urinary incontinence | “Mobile app Tἅt” vs wait list control for pelvic floor muscle training | Not peer reviewed, conference proceeding or presentation | Abstract included in Clinical Trials registry |
| Asklund et al., 2017 | Urinary incontinence | “Mobile app Tἅt” vs wait list control for pelvic floor muscle training | Intervention more than just initial contact-ongoing intervention contact not generated automatically | Non-respondents were reminded twice by email after 2 and 4 weeks and once by telephone after 6 weeks. |
| Avila et al., 2019 | Coronary arterial disease | Telemonitoring/rehab vs prolonged Cardiac rehabilitation vs Usual Care | Intervention more than just initial contact-ongoing intervention contact not generated automatically | Once a week, participants received feedback by phone or e-mail. |
| Backer et al., 2021 | Knee osteoarthritis – total knee arthroplasty | GenuSport app with knee trainer | Intervention more than just initial contact-ongoing intervention contact not generated automatically | Both groups followed a standardized identical postoperative protocol and identical pain management. |
| Bae et al., 2021 | Coronary heart disease | 1-way SMS text messaging program | Intervention more than just initial contact-ongoing intervention contact not generated automatically | Participants in the intervention group received 4 messages per week for 24 weeks in addition to standard care. |
| Ballin et al., 2020 | Central obesity | Web-based exercise vs wait list control | Participants are not adults with a chronic condition | BMI included people below 30 kg/m2-Did not fit with inclusion criteria |
| Bantum et al., 2014 | Cancer | Self-management programme for cancer with content on web-based material focussing on healthy behaviour that was supported by facilitators | Intervention more than just initial contact-ongoing intervention contact not generated automatically | Sessions were supported by facilitators who provided feedback and help. |
| Barnes et al., 2016 | Chronic obstructive pulmonary disease | Web based pulmonary rehabilitation over 7 weeks | Conference proceeding or presentation |  |
| Bateman et al., 2020 | Obesity and Overweight | FunForWellness online intervention | Participants are not adults with a chronic condition | The BMI criterion in this study includes overweight (i.e., 25.00–29.99 kg/m2) and obese (i.e., ≥ 30.00 kg/m2) categories. BMI included people below 30 kg/m2-Did not fit with inclusion criteria |
| Beerthuiszen et al., 2020 | Asthma | Internet-based self-management of severe asthma vs usual care | Intervention more than just initial contact-ongoing intervention contact not generated automatically | The helpdesk was accessible for information and communication technology purposes. |
| Bentley et al., 2020 | Chronic obstructive pulmonary disease | Smartphone app and activity tracker | Intervention more than just initial contact-ongoing intervention contact not generated automatically | The app was used initially in conjunction with the pulmonary rehab (PR) program, with continued use once the PR program had finished. |
| Berglind et al, 2020 | Mobility disability | App-based intervention using commercially available apps vs supervised health programme | Intervention more than just initial contact-ongoing intervention contact not generated automatically  AND  Participants are not adults with a chronic condition | The intervention included three face-to-face consultations, in groups of approximately 20 participants, where information on how to use the apps (session one at baseline), goal settings (session two at six weeks), and motivation to continue exercise (session three at 12 weeks) were discussed. |
| Bettger et al., 2019 | Total knee arthroplasty | Virtual exercise rehabilitation in-home therapy | Intervention more than just initial contact-ongoing intervention contact not generated automatically | telerehabilitation with remote clinician oversight by a physical therapist |
| Bini et al., 2017 | Total knee arthroplasty | Asynchronous video exercises between patient and physiotherapist vs traditional physiotherapy | Intervention more than just initial contact-ongoing intervention contact not generated automatically | Ongoing contact with a supervising physiotherapist to progress the exercises, even though it was asynchronous. |
| Bonata et al., 2020 | HIV | Exercise with app vs exercise with no app | Intervention more than just initial contact-ongoing intervention contact not generated automatically | During the study, participants received general dietary advice by nutritional biologists and through the app, participants received a weekly training plan, including date, duration, and distance of training, and a notification was sent before each training session to remind the exercise prescription. Online database daily monitored by the sport scientist. |
| Bosak et al., 2010 | Metabolic syndrome | Web-based intervention to increase self-efficacy to overcome barriers. | Intervention more than just initial contact-ongoing intervention contact not generated automatically | The principal investigator provided standardized e-mail feedback to participants each week on the achievement of exercise goals. |
| Bourne et al., 2017 |  |  | Intervention more than just initial contact-ongoing intervention contact not generated automatically | Contact details of the research team were provided so that participants had a point of reference for any queries they had regarding the technology or any health concerns. |
| Bozorgi et al., 2021 | Hypertension | Mobile intervention | Intervention more than just initial contact-ongoing intervention contact not generated automatically | Visits occurred for both groups throughout intervention period |
| Buchan et al., 2020 | Obesity and overweight | Wearable plus smartphone and tailored exercise programme | Participants are not adults with a chronic condition  AND  Intervention more than just initial contact-ongoing intervention contact not generated automatically | Participants could interact with each other or access researcher and/or technical  advice if they had questions or concerns about the intervention. |
| Bughin et al. 2021 | Obesity | Mobile telerehabilitation on metabolic and rehabilitation outcomes | Intervention more than just initial contact-ongoing intervention contact not generated automatically | Intervention also included teleconsultations at 1 and 2 months. In addition, doctors also had access to a secure website with access to patient data. |
| Castellano-Tejedor et al., 2020 | Obesity | PRECIOUS system app with biofeedback vs control vs app + biofeedback + motivational interviewin | Intervention more than just initial contact-ongoing intervention contact not generated automatically | Research and clinical team interacted with participants regarding the use, problems, feelings, experiences, and everything arisen by the patient while using the system throughout the intervention period. |
| Chaplin et al., 2017 | Chronic obstructive pulmonary disease | Web-based (SPACE for COPD) vs conventional PR programme | Intervention more than just initial contact-ongoing intervention contact not generated automatically | Weekly contact between participants and team via email/phone for motivational interviewing, progression of exercise and answering queries. |
| Chiang et al., 2020 | Cardiometabolic multimorbidity | Telerehab with heart rate sensing clothing-communicated to cloud for monitoring | Intervention more than just initial contact-ongoing intervention contact not generated automatically | Online communication was enabled using LINE software, which provided a platform for participants and researchers to interact with each other through messaging.  Exercise offered by a rehabilitation physician after the graded exercise testing and monitored by a physiotherapist and the nurse/researcher.  Weekly reminders and support from nurse. |
| Choi et al., 2019 | Frozen shoulder | Smartphone application vs conventional rehab | Intervention more than just initial contact-ongoing intervention contact not generated automatically | A clinical assistant contacted participants between scheduled visits to reduce the drop-out rate. |
| Chung et al., 2020 | Breast cancer | WalkON walking and Distress Thermometer | Not RCT powered to look at effectiveness-used data from previous study for control |  |
| Claes et al., 2020 | Cardiac | CR: PATHway | Intervention more than just initial contact-ongoing intervention contact not generated automatically | Four orientations sessions as part of continuation of cardiac rehabilitation. |
| Clays et al., 2021 | Congestive heart failure | HeartMan mobile personal health system for self-management | Intervention more than just initial contact-ongoing intervention contact not generated automatically | Patients in the intervention group continued to receive usual care, and additionally used the HeartMan personal health system in their home setting. |
| Connelly et al., 2017 | Type 2 diabetes living rurally | Interactive web group vs Info only web group vs leaflets containing web material | Intervention more than just initial contact-ongoing intervention contact not generated automatically | ‘ask the expert’ web feature however this is not made explicit as there is not a clear section on the intervention details (after the development). |
| Cramer et al., 2019 | Stroke | To increase arm therapy dose | Intervention more than just initial contact-ongoing intervention contact not generated automatically | Participants in the intervention group also received therapist feedback on supervised days, based on the therapist’s videoconference producing or presentation observations plus the therapist’s review of electronic data (prior days’ use, scores, and photographs during game play) and also received feedback on all days during game play. |
| Crawford et al., 2021a | Knee osteoarthritis-Total knee arthroplasty | Smartphone-based care platform vs traditional in-person physiotherapy rehabilitation | Intervention more than just initial contact-ongoing intervention contact not generated automatically | The smartphone-based care management system allowed messaging communication with the provider’s office in a text  messaging-like  application. |
| Crawford et al., 2021b | Knee osteoarthritis-total knee arthroplasty | Smartphone-based care platform vs traditional in-person physiotherapy rehabilitation | Additional publication from same study |  |
| Del Pozo et al., 2012 | Low back pain | Web-based exercise and postural education vs usual care | Participants are not adults with a chronic condition | LPB was over 6 weeks but less than 12 weeks. |
| Dorion-Cadrin et al., 2020 | Total knee and total hip arthroplasties | Tele-Prehabilitation vs usual prehabilitation | Intervention more than just initial contact-ongoing intervention contact not generated automatically | The tele-prehabilitation group performed the supervised exercise protocol at home. Only the first session was in person and the supervision of the home-based program  was provided by a physiotherapist through telecommunication applications (two sessions per week). |
| Duan et al., 2018 | Coronary heart disease | Web-based intervention to improve physical activity and fruit and vegetable consumption | Intervention more than just initial contact-ongoing intervention contact not generated automatically | To boost the engagement of patients, short message service (SMS) text messages were sent as reminders. Furthermore, the nurse contacted participants via phone calls once per week before each intervention session and at the 2 measurement points to remind the patients. Patients were also offered telephone cards as incentives for participation and data completion. |
| Duncan et al., 2020 | Overweight and obese | Smartphone app with education/BCT, face to face, Fitbit, scales vs Enhanced vs Traditional | Participants are not adults with a chronic condition  AND  Intervention more than just initial contact-ongoing intervention contact not generated automatically | BMI between 25 and 40 kg/m2. BMI included people below 30 kg/m2-Did not fit with inclusion criteria |
| Eichler et al., 2019 | Total knee arthroplasty | 3-month telerehabilitation as supplement and return to work using Kinect and synchronous or asynchronous communication with therapist | Intervention more than just initial contact-ongoing intervention contact not generated automatically | Three ways to communicate with therapist: participant records message for therapist to get, therapist to record message for participant to get when exercising, real time video communication during pre-determined exercise. |
| Ellis et al., 2019 | Parkinson’s disease | MHealth (Wellpepper app) exercise programme and walking programme vs pedometer and exercise programme only | Intervention more than just initial contact-ongoing intervention contact not generated automatically | Exercises adapted remotely and physiotherapist remotely monitored data, adherence, difficulty pain each week. Physiotherapist contacted participants via text message as needed. |
| Engelen et al., 2020 | Cardiovascular disease | Vascular View containing 6 modules | Not an RCT powered to look at effectiveness or is a protocol |  |
| Ferrante et al., 2017 | Cancer | Web based intervention plus Fitbit plus support | Not an RCT powered to look at effectiveness or is a protocol |  |
| Flachenecker et al., 2020 | Multiple sclerosis | Home internet-based PA promotion vs no intervention | Intervention more than just initial contact-ongoing intervention contact not generated automatically | Web- and telephone-based, behavior-oriented PA coaching with one individual and four group sessions, and individual exercise prescription in a one-to-one approach using a specialized, browser-based software solution. |
| Fleischman et al., 2019 | Total knee arthroplasty | Home exercises web-based vs printed manual (vs conventional clinic based) | Intervention more than just initial contact-ongoing intervention contact not generated automatically | Web-based intervention had interactive participant monitoring and communication portal. |
| Forbes et al., 2015 | Cancer | Web-Based PA programme vs usual care | Intervention more than just initial contact-ongoing intervention contact not generated automatically | Emails were developed to offer encouragement on PA-tailored if enough or not enough PA. |
| Frederix et a., 2015 | Coronary artery disease | Internet-based telerehab | Conference proceeding or presentation |  |
| Frensham et al., 2018 | Cancer | Pedometer + Website vs pedometer only | Intervention more than just initial contact-ongoing intervention contact not generated automatically | Researchers use inputs from participants to generate individually tailored target steps/day for the following week. |
| Frevel et al., 2015 | Multiple sclerosis | Internet-based home training programme vs hippotherapy | Intervention more than just initial contact-ongoing intervention contact not generated automatically | Therapist individually supervised every training session of each participant and adjusted the training schedules individually. |
| Ghadimi et al., 2021 | Chronic obstructive pulmonary disease | Telerehabilitation | Full next not in English |  |
| Ginis et al., 2016 | Parkinson’s disease | CuPiD gait training vs active control-personalised gait advice | Intervention more than just initial contact-ongoing intervention contact not generated automatically | CuPiD group participants received weekly home visits from the researcher during the six-week intervention. |
| Glasgow et al., 2010 | Diabetes | CASM (self-administered Computer Assisted Self-Management using social ecological model and 5As self-management model) vs CASM + SS (CASM with enhanced Social Support with follow up phone calls, group visit. | Intervention more than just initial contact-ongoing intervention contact not generated automatically | A moderated forum was used. |
| Gohir et al., 2021 | Knee osteoarthritis | iBEAT-OA internet-based treatment vs routine self-management | Intervention more than just initial contact-ongoing intervention contact not generated automatically | Adherence was encouraged by daily emails or smartphone notifications, or by the physiotherapist via asynchronous chat or telephone during the study period. |
| Golsteijn et al., 2018 | Prostate and colorectal cancer | OncoActive web-based intervention (PA advice tailored based on baseline info) vs usual care wait list | Intervention more than just initial contact-ongoing intervention contact not generated automatically | The option to consult a physical therapist for additional information is included. |
| Golsteijn et al., 2017 | Prostate and colorectal cancer | OncoActive web-based intervention (PA advice tailored based on baseline info) vs usual care wait list | Additional publication from same study |  |
| Gonzalez-Gerez et al., 2021 | Acute Covid | Telerehabilitation Program | Participants are not adults with a chronic condition | Participants had acute COVID-19 within last 40 days and no chronic conditions. |
| Grau-Pellicer et al., 2019 | Stroke | Multi-modal Rehab Program (MMRP) + mHealth app (Fitlab app) | Intervention more than just initial contact-ongoing intervention contact not generated automatically | Physical therapist guided sessions and WhatsApp group for motivation and to provide feedback, etc. |
| Grey et al., 2019 | Overweight and obese | Evolife website providing information about PA, healthy eating and behavioural changes using mismatch concept | Participants are not adults with a chronic condition AND  Intervention more than just initial contact-ongoing intervention contact not generated automatically | Participants were a combination of overweight and obese. BMI included people below 30 kg/m2-Did not fit with inclusion criteria and they met with researchers to discuss goals, go over what plans may look like, shown how to use pedometer, daily routines, etc. |
| Grobe et al., 2020 | Spinal cord injury | Internet-based exercise programme | Conference proceeding or presentation |  |
| Hageman et al., 2018 | Arthritis | Web-based weight loss intervention | Not an RCT powered to look at effectiveness or is a pilot paper without a control group |  |
| Hajizadeh et al., 2020 | Chronic obstructive pulmonary disease | TelePulmonary Rehab | Conference proceeding or presentation |  |
| Hansel et al., 2017 | Obesity and type 2 diabetes | ANODE intervention to help improve dietary habits and increase physical activity | Intervention more than just initial contact-ongoing intervention contact not generated automatically | Human contact provided to support in cases of technical issues |
| Hardt et al., 2018 | Knee osteoarthritis – total knee arthroplasty | GenuSport app with knee trainer | Intervention more than just initial contact-ongoing intervention contact not generated automatically | Both groups followed a standardized identical postoperative protocol and identical pain management. |
| Hassett et al., 2020 | Mobility limitation | Digital device as appropriate vs usual care | Participants are not adults with a chronic condition AND  Intervention more than just initial contact-ongoing intervention contact not generated automatically | Face-to-face and remote sessions following a health coaching model using phone, email, video conferencing, or in person at the participant’s discharge destination (home,  transitional living unit, residential care). |
| Hemmes et al., 2021 | Pulmonary arterial hypertension | Text-based mobile health intervention | Intervention more than just initial contact-ongoing intervention contact not generated automatically | All study participants, including those in the usual care arm, received daily reminders to sync their devices if more than 24 h had passed since the last syncing event. If no data were transmitted for more  than 48 h, the study team received an alert and contacted the participant to encourage compliance. |
| Hidrus et al., 2020 | Type 2 diabetes mellitus | WhatsApp exercise videos vs brochure on PA benefits | Intervention more than just initial contact-ongoing intervention contact not generated automatically | Participants reminded regularly as researchers uploaded an exercise video every week. |
| Hilmarsdottir et al., 2021 | Type 2 diabetes mellitus | Smartphone application (SidekickeHealth) vs control | Intervention more than just initial contact-ongoing intervention contact not generated automatically | The first author sent short, individualized encouragement through the app, based on registered activity in the app. After the first 16 weeks, both types of messages were received every other week for two more months. |
| Hiremath et al., 2019 | Spinal cord injury | PA promotion and PA with just in time prompts using smartwatch and wheel sensors | Not an RCT powered to look at effectiveness or is a pilot paper without a control group |  |
| Hochsmann et al., 2019 | Diabetes | Smartphone app with exergame using garden metaphor (+ consultation) vs one-time lifestyle counselling (+ consultation) | Intervention more than just initial contact-ongoing intervention contact not generated automatically | In both groups a sports medical expert provided a given number of personal exercise consultations on the telephone (weeks one and two). Consultations provided via telephone, including personal attention and instruction as well as technical support |
| Hou et al., 2019 | Low back pain | eHealth telehealth and website vs usual | Intervention more than just initial contact-ongoing intervention contact not generated automatically | Participants could receive daily reports about exercise and alerts to prompt them to return to this system. They could also communicate with their doctors through this system. Through the Web-based interface, the doctors could adjust rehab plans for participants and view reports about the participants’ daily exercise. |
| Houchen-Wolloff et al., 2018 | Cardiac rehabilitation | Web-based cardiac rehabilitation | Not an RCT powered to look at effectiveness or is a pilot paper without a control group |  |
| Ingram et al., 2019 | Unsure | e-coacherER | Conference proceeding or presentation |  |
| Isernia et al., 2019 | Chronic neurologic conditions | Human Empowerment Aging Disability (HEAD) on Patient Reported Outcome Measures (PROM) | Not an RCT powered to look at effectiveness or is a pilot paper without a control group |  |
| Jahangiry et al., 2017 | Metabolic syndrome | My Healthy Heart website interactive website vs control waiting list | Intervention more than just initial contact-ongoing intervention contact not generated automatically | Dietician sent tailored diet to participants’ inbox. Email reminders sent. Participants could ask their questions at any time and received responses within 24h. |
| Jennings et al., 2014 | Diabetes | Diabetes in Check website + pedometer vs control who received pedometer and limited website access | Intervention more than just initial contact-ongoing intervention contact not generated automatically | Participants in the intervention group were also distributed a weekly email reminder and encouraged to join discussions with programme manager. |
| Jiang et al., 2020 | Chronic obstructive pulmonary disease | WeChat PeR (pulmonary Internet Explorer Rehab) | Intervention more than just initial contact-ongoing intervention contact not generated automatically | Interaction with health professionals ongoing during intervention. |
| Jimenez-Reguera et al., 2020 | Chronic obstructive pulmonary disease | Happy Air mHealth Platform | Intervention more than just initial contact-ongoing intervention contact not generated automatically | Lovexair team provided tele-care support + monitoring throughout trial period. |
| Kanera et al., 2016 | Cancer | Kanker Nazorg Wijzer (Cancer Aftercare Guide, KNW) vs usual care | Intervention more than just initial contact-ongoing intervention contact not generated automatically | Research team monitored the forum to control for advice contradicting the advice given in the modules. |
| Kanera et al., 2017 | Cancer | Kanker Nazorg Wijzer (Cancer Aftercare Guide, KNW) vs usual care | Intervention more than just initial contact-ongoing intervention contact not generated automatically | Research team monitored the forum to control for advice contradicting the advice given in the modules. |
| Kannan et al., 2017 | Multiple sclerosis | Web-based fall prevention programme | Conference proceeding or presentation |  |
| Kannan et al., 2018 | Multiple sclerosis | Web-based fall prevention programme | Conference proceeding or presentation |  |
| Kannan et al., 2019 | Multiple sclerosis | Web-based fall prevention programme | Intervention more than just initial contact-ongoing intervention contact not generated automatically | Participants received a daily email directing them to a survey and were contacted by phone or email if they failed to respond to the fall survey for seven consecutive days. |
| Kayser et al., 2019 | Acute Coronary Syndrome | TAVIE en m@rche (videos delivered from virtual nurse based on profile determined from baseline online questionnaire) vs list of hyperlinks to increase daily steps | Intervention more than just initial contact-ongoing intervention contact not generated automatically | Non-automated management of reminders to the experimental group. Ability to contact team regarding health concerns |
| Kelechi et al., 2020 | Venous leg ulcers | FOOTFIT | Additional publication from same study |  |
| Kenfield et al., 2018 | Prostate cancer | Prostate 8 website + text messages vs control wait list | Conference proceeding or presentation |  |
| Kenfield et al., 2019 93 | Prostate cancer | Prostate 8 website + text messages vs control wait list | Intervention more than just initial contact-ongoing intervention contact not generated automatically | Every 2 weeks, participants  were emailed by the principal investigator about blog topics and recipes added on the website. |
| Ki et al., 2020 | Hemodialysis patients | Smartphone app exercises | Full next not in English AND Not an RCT powered to look at effectiveness or is a pilot paper without a control group |  |
| Kim et al., 2016 | Knee pain | SimpleTherapy web-video-based platform delivering progressive exercises based on user input vs web-based static exercises vs web-based video form | Intervention more than just initial contact-ongoing intervention contact not generated automatically | Clinicians monitored pain levels and feedback but did not directly communicate with participants except to answer email questions. |
| Kim et al., 2019 | Cardiovascular disease | mHeath using mobile app for 24 weeks for health behaviours | Intervention more than just initial contact-ongoing intervention contact not generated automatically | Participants received weekly individualized services related to healthy lifestyles. These services were provided by professional health management teams comprising physicians, nurses, nutritionists, and physical activity experts who monitored health information online in real-time. |
| Kim et al., 2020 | Hepatocellular cancer | mHealth Care App | Intervention more than just initial contact-ongoing intervention contact not generated automatically | Each participant was given an individualized  Rehabilitation exercise program that was  prescribed and adjusted at the 6-week mid-intervention period based on the assessment results. Participants’ conditions, as a result of their exercise programs, were communicated through the app’s real-time chat services. |
| Kloek et al., 2016 | Osteoarthritis | Blended intervention e-Exercise | Conference proceeding or presentation |  |
| Kloek et al., 2016 | Osteoarthritis | Blended intervention e-Exercise | Intervention more than just initial contact-ongoing intervention contact not generated automatically | Usual physical therapy sessions are integrated with a web‐based program. |
| Knudsen et al., 2019 | Ischemic heart disease or heart valve disease | Tele cardiac rehab | Intervention more than just initial contact-ongoing intervention contact not generated automatically | Patients were supervised through weekly tele-consultations (phone, email or text message). The consultation was based on weekly multi-disciplinary team conferences in which the overall rehabilitation  progress of each patient was evaluated. |
| Kolle et al., 2020 | Patellofemoral pain | Online exercise therapy plan (Mawendo GmbH) with video exercises, information, tips | Intervention more than just initial contact-ongoing intervention contact not generated automatically | Clarification sought-  No response from authors within required timeframe |
| Krein et al., 2013 | Low back pain | Pedometer upload weekly and study website with automated feedback, targeted messages, e-community vs usual care pedometer upload monthly | Intervention more than just initial contact-ongoing intervention contact not generated automatically | Research staff participated in and monitored the forum posts as well as used the forum to generate competitions to encourage meeting walking goals. |
| Lee et al., 2017 | Chronic neck pain | App with Mckenzie neck exercises and self-feedback on exercise record and pain | Intervention more than just initial contact-ongoing intervention contact not generated automatically | Both the app-based intervention group and the control group received text messages once a week to provide encouragement to the participants. |
| Lee et al., 2019 | Colorectal polyps | Noom Coach app + pedometer vs control (diary and newsletter). Both received monthly telephone calls | Intervention more than just initial contact-ongoing intervention contact not generated automatically | Both groups received monthly telephone calls for motivation |
| Lee et al. 2021 | Type 2 diabetes | Mobile application (LIBIT) for dietary and exercise management and Medilarm for medication adherence | Primary outcome is not physical activity or exercise related as per the ICF Framework | Outcomes of interest were changes in body weight, waist circumference, blood work values |
| Levinger et al., 2017 | Anterior cruciate ligament reconstruction | Internet interactive website vs usual post op care | Intervention more than just initial contact-ongoing intervention contact not generated automatically | Participants in both groups received usual care |
| Li et al., 2020 | Inpatient rehab patients | Pt Pal app-based exercise prog + rehab vs usual rehab | Intervention more than just initial contact-ongoing intervention contact not generated automatically | Intervention was in addition to ongoing in-person rehab |
| Lim et al., 2016 | Diabetes | U(ubiquitous) health care system and clinical decision support (CDSS) rule engine and PA monitoring device and dietary feedback vs Routine self-monitoring of blood glucose | Intervention more than just initial contact-ongoing intervention contact not generated automatically | Clarification sought-  No response from authors within required timeframe |
| Lison et al., 2020 | Hypertension and obesity | Live Well website | Intervention more than just initial contact-ongoing intervention contact not generated automatically | Researchers contacted participants if they stopped accessing the modules for more than two weeks after they were posted. In this case, a reminder email was sent. After three weeks without intervention access, a reminder telephone was made. |
| Liu et al., 2013 | Chronic obstructive pulmonary disease | Dyspnoea breathing programme with video instruction vs control | Intervention more than just initial contact-ongoing intervention contact not generated automatically | Participants who had not logging onto the online program regularly would receive a reminder by telephone from the respiratory nurse. |
| Loohuis et al., 2019 | Urinary incontinence | App-based treatment | Conference proceeding or presentation |  |
| Loohuis et al., 2021 | Urinary incontinence | URinControl app-based treatment | Intervention more than just initial contact-ongoing intervention contact not generated automatically | The research team provided technical support only. |
| Lozano Lozano et al., 2020 | Breast Cancer | BENECA mobile app | Intervention more than just initial contact-ongoing intervention contact not generated automatically | The CUIDATE group will make the telephone calls and send messages of encouragement. |
| Lunde et al., 2020 | Cardiac rehab | App vs usual care | Intervention more than just initial contact-ongoing intervention contact not generated automatically | Supervisor monitored and provided tailored feedback through app and email. Participants could submit questions and receive answers. |
| Maresca et al., 2019 | Aphasia | Virtual reality rehabilitation system (using a Tablet) | Intervention more than just initial contact-ongoing intervention contact not generated automatically | Twice a week, the neuropsychologist  performed a videoconference or presentation with patients to monitor the rehabilitation process carried out in their own home and discuss the feasibility and performance of the exercises. |
| Martin et al., 2015 | At risk of cardiovascular disease | Automated smart texts vs no texts | Participants are not adults with a chronic condition | Outpatients at an academic prevention centre but participants don’t all explicitly have a defined chronic health condition as part of the inclusion criteria |
| Mayberry et al., 2021 | Type 2 diabetes | REACH + FAMS vs REACH only | Intervention more than just initial contact-ongoing intervention contact not generated automatically | All participants—including those assigned to  the control condition—received access to a study helpline for questions about the study and their diabetes medications (a clinical pharmacist returned calls), text messages advising how to access study A1c results, and quarterly newsletters on healthy living with diabetes. |
| Mayer et al., 2018 | Colon cancer | CHESS smartphone app + smartphone vs National Cancer Institute booklet, survival toolbox and pedometer. | Intervention more than just initial contact-ongoing intervention contact not generated automatically | Staff facilitator and a personal trainer available for participants to ask questions about PA and to be involved in discussion group and to tailor messages to inactive participants |
| McConnon et al., 2007 | Obesity | Online advice, tools, information for behaviour change with personalised advice | Intervention more than just initial contact-ongoing intervention contact not generated automatically | Clarification sought-  No response from authors within required timeframe |
| McNeil et al., 2019 | Breast cancer | Activity trackers | Intervention more than just initial contact-ongoing intervention contact not generated automatically | Goals and barriers and strategies recorded to facilitate discussion by phone or email with study exercise physiologist to reinforce adherence, discuss problems barriers to achieve PA goals. |
| Morrison et al., 2016 | Asthma | RAISIN (randomized trial of asthma internet self-management intervention) Living with Asthma website vs usual care | Intervention does not explicitly involve self-management of PA or exercise | Study aims do not include any PA or exercise related aims. |
| Moy et al., 2015 | Chronic obstructive pulmonary disease | Taking Healthy Steps (internet mediated, pedometer-based walking programme) vs wait list control with pedometer only | Intervention more than just initial contact-ongoing intervention contact not generated automatically | All participants who were randomized to the  intervention arm had access to the study staff for questions, which could be initiated by sending an email or directly on the website through a form. Participants could also call the staff on a toll-free number. Study staff usually responded with a phone call. |
| Muller et al., 2017 | Diabetes | Healthy living with diabetes-web-based interactivity v web-based plain text | Primary outcome is not physical activity or exercise related as per the ICF Framework | Powered to assess change in outcomes of Usage, engagement, health literacy |
| Murphy et al., 2021 | Cutaneous systemic sclerosis | Intensive and app-delivered occupational therapy | Intervention more than just initial contact-ongoing intervention contact not generated automatically | The therapist checked on progress via dashboard, communicated with the participant as needed, and uploaded  other information (such as digital ulcer management and home paraffin wax treatment) weekly on the App for all  participants. |
| Myers et al., 2020 | Obesity and overweight | Fun For Wellness (FFW) vs Usual care | Participants are not adults with a chronic condition | The BMI criterion included people below 30 kg/m2-Did not fit with inclusion criteria. |
| Nelligan et al., 2021 | Knee osteoarthritis | Self-directed web-based strengthening exercises and physical activity program supported by automated text messages | Intervention more than just initial contact-ongoing intervention contact not generated automatically | Certain text scenarios e.g ‘Response not supported: “Encourages contacting research team if needed. Inappropriate responses are monitored by the research team. Any that require action (e.g. safety concern) will be followed up by the research team. |
| Nyberg et al., 2019 | Chronic obstructive pulmonary disease | COPD-web (with condition + professionals working with those with the condition) + usual care | Intervention more than just initial contact-ongoing intervention contact not generated automatically  AND  Participants are not adults with a chronic condition | Healthcare professional section of the website aimed at facilitating the *provision of support* for self-management strategies for people with COPD, thereby facilitating the implementation of such services and reinforcing the use of the COPD-web during follow-up visits |
| Ormel et al., 2018 | Cancer | Runkeeper app self-monitoring of PA with training reminder vs usual care of advice to be PA | Intervention more than just initial contact-ongoing intervention contact not generated automatically | One investigator was available for answering questions about the RunKeeper use by telephone or e-mail. |
| Ozen et al., 2021 | Stroke | Computer game assisted task specific exercises (CGATSE) for arm function vs standard occupational therapy | Intervention more than just initial contact-ongoing intervention contact not generated automatically | Those allocated to the CGATSE  group, received thirty minutes of CGATSE using the Rehabilitation Joystick for Computerized Exercise (Rejoyce) system five days per week under the supervision of the same occupational therapist in addition to one hour of PT |
| Pagliari et al., 2021 | Multiple sclerosis | Integrated telerehabilitation using virtual reality vs home-based conventional rehabilitation | Intervention more than just initial contact-ongoing intervention contact not generated automatically | The exercises were performed at home by the patient using a dedicated virtual reality rehab system home-based kit in an asynchronous telerehab modality  with digital contents and offline remote monitoring by the therapist |
| Park et al., 2020 | Chronic obstructive pulmonary disease | SASMP (smart app based self-management program) on self-care for COPD | Intervention more than just initial contact-ongoing intervention contact not generated automatically | Participants were encouraged to communicate with other participants and research team by text messages in the smartphone app or call. |
| Passalent et al., 2016 | Anxial spondyloarthritis | Interactive web-based e-Learning education module | Conference proceeding or presentation |  |
| Passalent et al., 2017 | Anxial spondyloarthritis | Interactive web-based e-Learning education module | Conference proceeding or presentation |  |
| Petrozzi et al., 2019 | Low back pain and chronic pain | Physiotherapy +/- MoodGYM | Intervention NOT explicitly directed to PA/ex  AND  Intervention more than just initial contact-ongoing intervention contact not generated automatically | MoodGYM CBT focussed intervention on mood NOT on changing behaviour of PA or exercise and participants were contacted weekly by research staff to increase adherence. |
| Pfaeffli Dale et al., 2015 | Coronary heart disease | Text4Heart SMS and website 24-week intervention vs usual care | Intervention more than just initial contact-ongoing intervention contact not generated automatically | Individual questions were responded to personally by the research team within 48 hours |
| Plow et al., 2017 | Chronic health conditions | mHealth self-management via tablet and use of existing apps vs paper based self-management vs contact control | Intervention more than just initial contact-ongoing intervention contact not generated automatically | Participants received three follow-up phone calls at a frequency of one call placed every other week for six weeks after the in-person session |
| Rafiq et al., 2021 | Knee osteoarthritis | Lower limb rehabilitation with mobile health intervention | Intervention more than just initial contact-ongoing intervention contact not generated automatically | In addition, every patient was actively followed by phone at least once a week to ensure that they read the messages and performed the intervention. |
| Rees-Punia et al., 2021 | Cancer | Health and Energy through Active Living Every Day (HEALED) web-based PA vs as usual wait-listed control | Intervention more than just initial contact-ongoing intervention contact not generated automatically | HEALED intervention group participants received monthly motivational e-mails announcing one new website  feature to prompt return to the website |
| Reguera et al., 2017 | Chronic obstructive pulmonary disease | Integrated internet programme after pulmonary rehab vs conventional | Conference proceeding or presentation |  |
| Rigot et al., 2021 | Wheelchair users | One off transfer skills training intervention delivered over the internet | Intervention does not explicitly support self-management of physical activity or exercise in an interactive self-management programme | Intervention is skills training, not a physical activity or exercise programme. |
| Riva et al., 2014 | Low back pain | ONESELF website containing static (library, first aid, FAQ) and interactive (Virtual gym, action plan, testimonials, commentaries, quiz) features | Intervention more than just initial contact-ongoing intervention contact not generated automatically | Clarification sought-  No response from authors within required timeframe |
| Robinson et al., 2020 | Chronic obstructive pulmonary disease | Internet-mediated walking programme with goal setting, feedback, motivation and education messages, and social support | Conference proceeding or presentation |  |
| Robinson et al., 2021 | Chronic obstructive pulmonary disease | Internet-mediated walking programme with goal setting, feedback, motivation and education messages, and social support | Intervention more than just initial contact-ongoing intervention contact not generated automatically | Follow-up, in-person assessments occurred at 3 and 6 months for both groups. At each follow-up visit, study staff reminded participants not to disclose randomisation assignment and that they should be working to increase their walking and  exercise. |
| Salaffi et al., 2015 | Fibromyalgia | Multicomponent intervention and web-based evaluation | Intervention not web-based |  |
| Salaffi et al., 2020 | Fibromyalgia | Web-based intervention | Control data from previous work |  |
| Salerno et al., 2021 | Cancer | DVD delivered physical activity intervention | Intervention not web-based  Intervention more than just initial contact-ongoing intervention contact not generated automatically | Both conditions received titrated support telephone calls from intervention  staff over the course of the six months |
| Schweier et al., 2014 | Coronary heart disease and low back pain | Peer mentoring website containing clips on peoples’ experiences on successfully modified behaviour vs usual care | Intervention more than just initial contact-ongoing intervention contact not generated automatically | Participants encouraged to contact the project team in case of problems or questions and those who provided an email received email reminders. |
| Scorza et al., 2020 | Parkinson’s disease | Mhealth-supported exercise | Opinion article – not RCT |  |
| Simpson et al., 2020 | Obesity | HelpMeDoIt! Website and app providing evidence-based information on weight loss, goals, social support and methods on how to harness, set and monitor these using existing resources. | Intervention more than just initial contact-ongoing intervention contact not generated automatically | Clarification sought-  No response from authors within required timeframe |
| Shang-Lin et al., 2020 | Cardiometabolic multimorbidity | Home-based telehealth exercise training program | Intervention more than just initial contact-ongoing intervention contact not generated automatically | A weekly reminder for maintenance of exercise and providing patient support |
| Short et al., 2017 | Cancer | Web-based physical activity advice | Intervention more than just initial contact-ongoing intervention contact not generated automatically | Participants were sent up to two email reminders (3 days apart) each time they had a module due. Participants were also  sent up to two reminders to complete action plans when they became available |
| Smith et al., 2019 | Pain | Reboot Online with graded exercises and narrated videos of three movement stations: flexibility, strength, stability; resources, relaxation stations. | Intervention more than just initial contact-ongoing intervention contact not generated automatically | Participants were contacted via e-mail or  telephone by the research technician after the first two lessons, then as requested by the participant. |
| Snoeck-Stroband et al., 2017 | Asthma | Tailored web-based self-management support (SMS) in addition to standard care | Conference proceeding or presentation |  |
| Streber et al., 2018 | Multiple sclerosis | Internet-based after-care program | Conference proceeding or presentation |  |
| Tallner et al., 2012 | Multiple sclerosis | At home physical training (e-training) protocol focused on strength and endurance | Conference proceeding or presentation  Intervention more than just initial contact-ongoing intervention contact not generated automatically | Training protocols were supervised and adjusted by sports therapists. |
| Tarakci et al., 2021 | Multiple sclerosis | 12 week structured telerehabilitation program vs 12 week structured in person exercise program | Intervention more than just initial contact-ongoing intervention contact not generated automatically | Patients in Group 2 were checked and controlled by means of telerehabilitation three times per week. Patients received telephone video calls to control their adherence to the exercise sessions and  revise their exercises if needed |
| Taylor et al., 2020 | Chronic conditions | ecoachER | Intervention more than just initial contact-ongoing intervention contact not generated automatically | Participants in the intervention group continued to receive usual ERS input. |
| Taylor et al., 2020 | Chronic conditions | ecoachER | Additional publication from same study |  |
| Thielbar et al., 2020 | Stroke | Virtual Reality | Intervention more than just initial contact-ongoing intervention contact not generated automatically | Therapy involved Multi-User which involved therapist + participant (therapist in clinic + participant at home) in real time. |
| Thiengwittayaporn et al., 2021 | Knee osteoarthritis | Mobile application | Intervention more than just initial contact-ongoing intervention contact not generated automatically | Clarification sought-  No response from authors within required timeframe |
| Tomita et al., 2019 | Heart failure | Information and exercise support with video | Intervention more than just initial contact-ongoing intervention contact not generated automatically | Appraisal support was provided via email every month from appropriate health care professionals in dealing with subjects for the purpose of feedback for participants’ records. This support was provided to encourage participant’s ongoing actions for changing their health behaviors toward a maintenance stage (beyond six months). Emotional support was provided on an as needed basis via email. |
| Uhm et al., 2017 | Breast cancer | Mhealth + pedometer vs usual care | Intervention more than just initial contact-ongoing intervention contact not generated automatically | Clarification sought-  No response from authors within required timeframe |
| Ulcoq et al., 2019 | Knee arthroscopy | Internet based physiotherapy | Conference proceeding or presentation |  |
| Van de Wiel et al., 2021 | Breast and prostate cancer survivors | Internet based physical activity support program | Intervention more than just initial contact-ongoing intervention contact not generated automatically | Program involved physiotherapist telephone counselling |
| Van den Berg et al., 2006 | Rheumatoid arthritis | Internet-based PA interventions (individualised) vs generalised | Intervention more than just initial contact-ongoing intervention contact not generated automatically | Every week the participants sent back a completed program schedule by e-mail, and subsequently a new schedule was put on the participant’s personal web page. Participants received weekly, individual distant supervision by e-mail from 2 experienced physical therapists. |
| Van Dijk-Huisma et al., 2020 | Post op total knee or hip arthroplasties | MOX sensor + Hospital Fit App to increase PA levels vs usual care | Intervention more than just initial contact-ongoing intervention contact not generated automatically | The extent of functional recovery was evaluated by the physiotherapist during every treatment. |
| Van Reijen et al., 2017 | Ankle sprains | Strengthen your ankle app with videos and verbal instructions of 6 exercises with progressions to increase weekly over 8 weeks + balance board vs paper-based version + balance board | Intervention more than just initial contact-ongoing intervention contact not generated automatically | Participants received a request by email to complete an online compliance questionnaire. After 3 days, a reminder was sent in case of non-response |
| Vluggen et al., 2021 | Type 2 Diabetes | Web-based computer tailored program | Intervention more than just initial contact-ongoing intervention contact not generated automatically | The program is self-guided and facilitated through periodic prompts and  reminders to stimulate program engagement and completion. |
| Volders et al., 2020 | Older adults with chronic illness | Active Plus intervention vs wait list control | Intervention more than just initial contact-ongoing intervention contact not generated automatically | Participants in the intervention group received advice on three occasions, both online on a secured website and paper (via a letter by mail) that was based on their responses to questionnaires, participants’ characteristics, psychosocial stance, current PA, and what local PA was possible. |
| Volders et al., 2021 | Older adults with chronic illness | Active Plus intervention for older adults with chronic diseases | Intervention more than just initial contact-ongoing intervention contact not generated automatically | Participants receive personal advice by email and by mail (from protocol Volders et al., 2019) |
| Wadensten et al., 2021 | Urgency and mixed urinary incontinence in women | Mobile app for self-management of urgency and mixed urinary incontinence in women | Intervention more than just initial contact-ongoing intervention contact not generated automatically | If the app was not activated within 2 weeks, an email reminder was sent to the participant, and if it was not activated within another week, the participant was contacted via telephone and offered technical guidance. |
| Wan et al., 2017 | Chronic obstructive pulmonary disease | Every step counts (ESC) | Intervention more than just initial contact-ongoing intervention contact not generated automatically | Able to contact staff through website and participated in a minimum of two in-person clinic visits. |
| Wan et al., 2020 | Chronic obstructive pulmonary disease | Every Step Counts (ESC) | Secondary data to previous study |  |
| Wang, et al., 2020 | Chronic obstructive pulmonary disease | Mobile health app vs routine care | Intervention more than just initial contact-ongoing intervention contact not generated automatically | One app module mainly provided motivational support to participants, which included peer support chat room and an expert support portal. |
| Wang, et al., 2021 | Chronic obstructive pulmonary disease | Mobile application to support self-management | Intervention more than just initial contact-ongoing intervention contact not generated automatically | Clarification sought-  No response from authors within required timeframe |
| Wang, et al., 2020 | Urinary stress incontinence | Audio guidance pelvic floor muscle training program app where participants are guided by audio to contract and relax pelvic floor muscles | Intervention more than just initial contact-ongoing intervention contact not generated automatically | Significant input at baseline and researchers called the participants of the two groups once a month to answer their questions related to training or stress incontinence and encourage them to keep on training for at least three months. |
| Webb et al., 2019 | Cancer | Move More Pack printed components and internet tools and e-newsletters drawing on theory vs waitlist control | Inter more than just initial contact-ongoing intervention contact not generated automatically- | ‘Ask a physio’ component on the internet component. |
| Wu et al., 2020 | Stroke | Telerehabilitation video conferencing twice a week | Intervention more than just initial contact-ongoing intervention contact not generated automatically | The collaborative care team developed rehab plans and once the patient was discharge home from in-patient care, the rehabilitation engineer and rehabilitation nurse performed personalized remote rehabilitation instruction twice a week. |
| Yun et al., 2020 | Cancer | Health coaching through Smart Management Strategies for Health (SMASH) to improve health behaviors such as PA, weight control and distress management | Intervention more than just initial contact-ongoing intervention contact not generated automatically | Clarification sought-  No response from authors within required timeframe |
