# Supplementary figures and images for: The Effectiveness of Self-Guided Digital Interventions to Improve Physical Activity and Exercise Outcomes for People With Chronic Conditions: A Systematic Review and Meta-Analysis
Source: Front Rehabil Sci. 2022 Jun 24;3:925620. doi: 10.3389/fresc.2022.925620 (PMC9397696; doi:10.3389/fresc.2022.925620)

**Supplementary File 2.** Risk of Bias of included studies

**
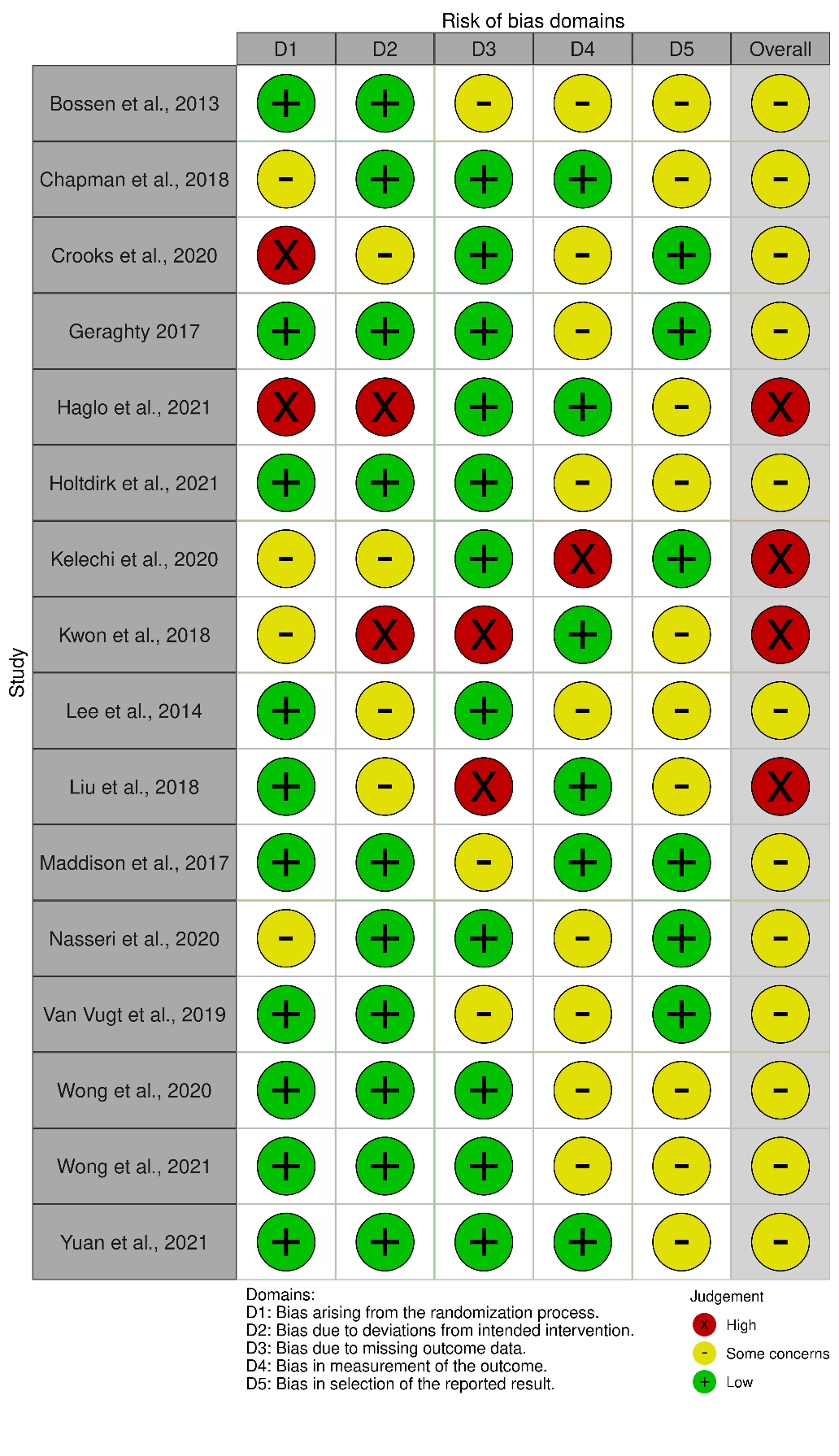
**

Supplement: Supplementary file 2 [file Data_Sheet_2.docx]
