# Supplementary material for: The Effectiveness of Self-Guided Digital Interventions to Improve Physical Activity and Exercise Outcomes for People With Chronic Conditions: A Systematic Review and Meta-Analysis
Source: Front Rehabil Sci. 2022 Jun 24;3:925620. doi: 10.3389/fresc.2022.925620 (PMC9397696; doi:10.3389/fresc.2022.925620)
Supplement: Supplementary file 3 [file Data_Sheet_3.docx]

**Supplementary File** **3**. Results across studies and outcome measures.

| Author | Outcome measure | | Assess point (weeks) | Mean group difference (Variance) | 95% confidence intervals | SD | Hedge’s g | 95% confidence intervals |
| --- | --- | --- | --- | --- | --- | --- | --- | --- |
|  | Self-report | Instrument |  |  |  |  |  |  |
| **Physical activity** | | | | | | | | |
| Bossen et al. 2013  **OA** | **PASE (0-400)** |  | 12 | -1.6 | -16.6 to 13.5 | 44 | -0.03 | -0.3 to 0.3 |
|  |  |  | 52 | 21.2 | 3.6 to 38.9 | 54.1 | 0.4 | 0.1 to 0.7 |
|  |  | **Physical activity via ActiGraph GT3X accelerometer (min/day)** | 12 | 3 | -26 to 32 | 11.1 | 0.1 | -0.5 to 0.6 |
|  |  |  | 52 | 24 | 0.5 to 46.8 | 43.1 | 0.6 | 0.001 to 1.1 |
| Chapman et al. 2018  **Breast cancer** | **LSI-MSPA (calculated score)** |  | 4 | 2.4 | -2.2 to 7.1 | 11.1 | 0.2 | -0.2 to 0.6 |
|  |  |  | 24 | 7.2 | 2.3 to 12.1 | 11.1 | 0.6 | 0.2 to 1.1 |
| Crooks et al. 2020 **COPD** |  | **7-day daily step count via Fitbit (number)** | 12 | -2252.9 | -10433.8 to 5927.9 | 5577.9 | -0.4 | -1.5 to 0.7 |
| Holtdirk et al. 2021  **Breast cancer** | **IPAQ-SF (MET-min/ week)** |  | 12 | 243 | NR | 2593 | 0.1 | -0.1 to 0.3 |
| Lee et al. 2014  **Breast cancer** | **7-day exercise diary (reporting minutes per week ≥ 4 METs)** |  | 12 | 3.4 | 1.2 to 10.2 | Odds ratio | 0.3 | -0.2 to 0.8 |
| Liu et al. 2018  **HTN** |  | **Daily step count via XL-18CN pedometer (number)** | **expert driven** | | | | | |
|  |  |  | 16 | 2460 | 1137 to 3783 | 2980.7 | 0.8 | 0.4 to 1.3 |
|  |  |  | **user driven** | | | | | |
|  |  |  | 16 | 2459 | NR | 2403.2 | 1.0 | 0.6 to 1.5 |
| Maddison et al. 2015  **IHD** | **IPAQ-LF (Median MET-min/ week)** |  | 24 | 233.9 | -146.5 to 614.2 | 1200.1 | 0.2 | -0.1 to 0.5 |
| Nasseri et al. 2020  **MS** | **LSI-MSPA (calculated score)** |  | 12 | -3.0 | -12.9 to 6.8 | 21.3 | -0.1 | -0.8 to 0.5 |
|  |  | **mod-vigorous PA via Actigraph accelero-meter (%)** | 12 | -2.9 | -7.8 to -1.9 | 10.5 | -0.3 | -0.9 to 0.4 |
| Wong et al. 2020  **CHD** | **GSLTPAS (calculated score)** |  | 12 | 3.6 | 1.0 to 6.1 | 13 | 0.3 | 0.1 to 0.5 |
|  |  |  | 24 | 2.3 | -0.4 to 5.2 | 12.5 | 0.2 | -0.02 to 0.4 |
| Wong et al. 2021  **Metabolic Syndrome** | **GSLTPAS (calculated score)** |  | 4 | 5.9 | NR | 16.3 | 0.4 | -0.1 to 0.8 |
|  |  |  | 12 | 8.6 | NR | 18 | 0.5 | 0.02 to 0.9 |
| **Body function and symptoms** | | | | | | | | |
| Bossen et al. 2013  **OA** | **KOOS / HOOS (0-100)** |  | 12 | 6.5 | 1.8 to 11.2 | 15.4 | 0.4 | 0.1 to 0.7 |
|  |  |  | 52 | 5.0 | -1.0 to 11.0 | 18.6 | 0.3 | -0.1 to 0.6 |
|  | **Self-perceived effect (1-7)** |  | 12 | 10.7 | 4.3 to 26.4 | Odds ratio | 0.56 | 0.3 to 0.9 |
|  |  |  | 52 | 1.2 | 0.6 to 2.4 | Odds ratio | 0.04 | -0.4 to 0.5 |
| Crooks et al. 2020 **COPD** | **CAT (0-40)** |  | 12 | -1.3 | -4.47 to -1.92 | 12.4 | 0.1 | -0.4 to 0.6 |
| Geraghty et al. 2017  **Dizziness** | **VSS-SF (0-60)** |  | 12 | -2.8 | -1.4 to -4.1 | 3.5 | 0.8 | 0.5 to 1 |
|  |  |  | 24 | -2.3 | -0.3 to -4.1 | 7.23 | 0.31 | 0.1 to 0.6 |
| Haglo et al. 2021 **Inflammatory rheumatic diseases** |  | **VO_2_ max via Metamax II (ml/kg/ min)** | 10 | 0.1 | NR | 6 | 0.02 | -0.6 to 0.6 |
| Kelechi et al. 2020  **Venous leg ulcers** |  | **ROM (df) of right ankle (degrees)** | 6 | -3.1 | NR | 8.6 | -0.35 | -1.1 to 0.4 |
|  |  | **ROM (df) of left ankle (degrees)** | 6 | -6.3 | NR | 9.3 | -0.65 | -1.46 to 0.16 |
|  |  | **Strength (pf) of right ankle (kg)** | 6 | -4.4 | NR | 9 | -0.5 | -1.3 to 0.3 |
|  |  | **Strength (pf) of left ankle (kg)** | 6 | -1.8 | NR | 8.8 | -0.2 | -0.1 to 0.6 |
|  | **FAAM (0-100)** |  | 6 | -0.7 | NR | 30.8 | -0.02 | -0.8 to 0.8 |
|  |  | **6MWT (m)** | 6 | -18 | NR | 544.7 | -0.03 | -0.8 to 0.8 |
| Kwon et al. 2018  **COPD** |  | **6MWT^a^ (m)** | **Fixed-interactive** | | | | | |
|  |  |  | 6 | -8.7 | NR | 94.54 | -0.1 | -0.6 to 0.4 |
|  |  |  | 12 | -6.6 | NR | 82 | -0.1 | -0.7 to 0.5 |
|  |  |  | **Fixed** | | | | | |
|  |  |  | 6 | 10.9 | NR | 70.5 | 0.15 | -0.5 to 0.8 |
|  |  |  | 12 | 10.3 | NR | 73.4 | 0.14 | -0.5 to 0.8 |
|  | **CAT^a^ (0-40)** |  | **Fixed-interactive** | | | | | |
|  |  |  | 6 | -3.1 | NR | 8.6 | 0.4 | -0.2 to 0.9 |
|  |  |  | 12 | -0.3 | NR | 9 | 0.03 | -0.5 to 0.6 |
|  |  |  | **Fixed** | | | | | |
|  |  |  | 6 | -4 | NR | 8.1 | 0.5 | -0.1 to 1.1 |
|  |  |  | 12 | -1.4 | NR | 9.5 | 0.1 | -0.5 to 0.8 |
|  | **mMRC^a^ (0-4)** |  | **Fixed-interactive** | | | | | |
|  |  |  | 6 | -0.01 | NR | 0.8 | 0.01 | -0.5 to 0.6 |
|  |  |  | 12 | 0.1 | NR | 0.8 | -0.1 | -0.7 to 0.5 |
|  |  |  | **Fixed** | | | | | |
|  |  |  | 6 | -0.2 | NR | 0.8 | 0.2 | -0.2 to 0.9 |
|  |  |  | 12 | -0.1 | NR | 0.7 | 0.2 | -0.5 to 0.6 |
| Van Vugt et al. 2019  **Dizziness** | **VSS-SF (0-60)** |  | 12 | -4.3 | -5.9 to -2.6 | 5.8 | 0.7 | 0.5 to 1.0 |
|  |  |  | 24 | -4.1 | -5.8 to -2.5 | 5.8 | 0.7 | 0.4 to 1.0 |
| Maddison et al. 2015  **IHD** |  | **Peak VO_2_ via Moxus (ml/kg/ min)** | 24 | -0.2 | -1.1 to 0.7 | 2.8 | -0.1 | -0.4 to 0.2 |
| Wong et al. 2020 **CHD** | **SEE (0-90)** |  | 12 | 0.3 | -12 to 0.6 | 2.1 | 0.1 | -0.1 to 0.3 |
|  |  |  | 24 | -0.03 | -0.4 to 0.2 | 2.2 | -0.02 | -0.2 to 0.2 |
| Wong et al. 2021  **Metabolic Syndrome** | **SEE (0-90)** |  | 4 | 8.1 | NR | 15.6 | 0.5 | 0.1 to 1 |
|  |  |  | 12 | 9.9 | NR | 15.9 | 0.6 | 0.2 to 1.2 |
| Yuan et al. 2021 **Fibromyalgia** | **WPI (0-19)** |  | 6 | 0.2 | -2.3 to 2.8 | 4.3 | -0.05 | -0.7 to 0.6 |
|  | **VAS (0-10)** |  | 6 | -0.4 | -1.3 to 2.2 | 2.4 | 0.2 | -0.4 to 0.8 |
|  | **SS (0-12)** |  | 6 | -0.2 | -1.5 to 1.9 | 2.5 | 0.1 | -0.5 to 0.7 |

**4 MET** moderate aerobic exercise that consumed at least 3.5 ml/O_2_/kg/min or > 150 min/week, **6MWT** six minute walk test, **AROM** active range of motion, **BP** blood pressure, **CAT** COPD assessment test, **df** dorsiflexion, **FAAM** Foot and Ankle Mobility Measure, **GSLTPAS** Godin Shepherd Leisure Time Physical Activity Scale, **HOOS** Hip Osteoarthritis Outcome Score, **KOOS** Knee Osteoarthritis Outcome Score, **kg** Kilogram, **LSI** Leisure Score Index, **LSI-MSPA** moderate-strenuous exercise, **LSI-Mild** light exercise, **m** meter, **mm/Hg** millimeters of mercury, **mMRC** Modified Medical Research Council Dyspnea Scale, **NR** not reported, **PASE** Physical Activity Scale for the Elderly, **pf** plantarflexion, **ROM** range of motion, **SD** standard deviation, **SS** severity symptom scale, **SEE** Self-efficacy for exercise, **VO_2_** maximum rate of oxygen consumption, **VAS** visual analogue scale, **VSS-SF** vestibular symptom scale-short form, ^a^ data taken from figures using WebPlotDigitizer (31), **WPI** Widespread pain index
